# Evaluating the Outcomes of the Menthol Cigarette Ban in England by Comparing Menthol Cigarette Smoking Among Youth in England, Canada, and the US, 2018-2020

**DOI:** 10.1001/jamanetworkopen.2022.10029

**Published:** 2022-05-03

**Authors:** Katherine A. East, Jessica L. Reid, Robin Burkhalter, Loren Kock, Andrew Hyland, Geoffrey T. Fong, David Hammond

**Affiliations:** 1 School of Public Health Sciences, Faculty of Health, University of Waterloo, Waterloo, Ontario, Canada; 2 National Addiction Centre, Institute of Psychiatry, Psychology and Neuroscience, King’s College London, London, United Kingdom; 3 Department of Behavioural Science and Health, University College London, London, United Kingdom; 4 Department of Health Behavior, Roswell Park Comprehensive Cancer Center, Buffalo, New York; 5 Department of Psychology, University of Waterloo, Waterloo, Ontario, Canada; 6 Ontario Institute for Cancer Research, Toronto, Ontario, Canada

## Abstract

**Importance:**

Menthol cigarettes were prohibited in England in May 2020 and nationally in Canada in October 2017 but remain permitted in the US. Evidence on the outcomes of menthol cigarette bans among youth outside of Canada, and the characteristics of youth smokers, is lacking.

**Objectives:**

To evaluate the outcomes of menthol cigarette bans on youth menthol cigarette smoking and to characterize youth menthol cigarette smokers in terms of demographics and cigarette consumption and dependence.

**Design, Setting, and Participants:**

This survey study uses data from online repeat cross-sectional International Tobacco Control Youth Tobacco and Vaping Surveys conducted in 2018, 2019, February 2020, and August 2020. Participants included past 30-day smokers aged 16 to 19 years. Data analysis was performed from March 2021 to January 2022.

**Main Outcomes and Measures:**

Usually smoke a brand of cigarettes that was menthol, including capsule.

**Exposures:**

Menthol cigarette ban, comparing 3 countries over time: Canada, where a ban already existed, England, where a ban was implemented during the study, and the US, where no national ban was present. Age, sex, race, and consumption and dependence were also examined by menthol smoking in each country, and in England before vs after the ban.

**Results:**

The analytical sample comprised 7067 participants aged 16 to 19 years, of whom 4129 were female and 5019 were White. In England, the weighted percentage of youth smokers who reported smoking a menthol or capsule cigarette brand was stable in the 3 survey waves before the menthol ban (2018 to February 2020, 9.4% vs 12.1%; adjusted odds ratio [AOR], 1.03; 95% CI, 0.99-1.06; *P* = .15) but decreased to 3.0% after the ban (February 2020 vs August 2020, AOR, 1.07; 95% CI, 1.04-1.10; *P* < .001). The decrease between February and August 2020 in England was similar across all demographic groups but was greater among youth who perceived themselves as addicted to cigarettes (AOR, 0.37; 95% CI, 0.41-0.97; *P* = .04). In the 2 comparison countries, menthol or capsule smoking was stable across all waves (2018 to August 2020: US, 33.6%-36.9%; Canada, 3.1%-2.3%) and was more prevalent in the US than in England (AOR, 5.58; 95% CI, 4.63-6.72; *P* < .001). Menthol or capsule smoking was also more prevalent among smokers in England who were female vs male (10.9% vs 7.2%; AOR, 1.04; 95% CI, 1.01-1.06; *P* = .002); among smokers in the US who identified as Black vs White (60.6% vs 31.9%; AOR, 1.33; 95% CI, 1.23-1.44; *P* < .001) or who were frequent smokers (AOR, 1.07; 95% CI, 1.01-1.13; *P* = .03), smoked more cigarettes per day (2-5 vs 1, AOR, 1.09; 95% CI, 1.02-1.15; *P* = .006; >5 vs 1, AOR, 1.10; 95% CI, 1.03-1.18; *P* = .007), or had urges to smoke every or most days (AOR, 1.08; 95% CI, 1.02-1.14; *P* = .006); and among smokers in Canada who perceived themselves as addicted to cigarettes (AOR, 1.02; 95% CI, 1.00-1.03; *P* = .01).

**Conclusions and Relevance:**

In this survey study, the proportion of youth smokers who smoke menthol (including capsule) cigarettes decreased substantially after the menthol ban in England. This association was consistent across all demographic groups. Perceived addiction among menthol smokers was also lower where menthol cigarettes were banned.

## Key Points

**Question:**

How was the menthol cigarette ban in England associated with menthol cigarette smoking among youth smokers?

**Findings:**

This survey study of 7067 youth smokers found that the menthol cigarette ban in England was associated with a significant reduction in the prevalence of menthol cigarette smoking, from 12.1% to 3.0%. Menthol cigarette smoking remained stable in the 2 comparison countries, Canada (3.1%-2.3%) and the US (33.6%-36.9%), and the association was consistent across all demographic groups.

**Meaning:**

These findings suggest that the proportion of youth smokers who smoke menthol (including capsule) cigarettes decreased substantially following the menthol ban in England.

## Teaser

This survey study evaluates the outcomes of menthol cigarette bans on youth menthol cigarette smoking and characterizes youth menthol cigarette smokers in terms of demographics and cigarette consumption and dependence.

## Introduction

Menthol is added to tobacco products to improve taste and appeal and to reduce harshness and perceived harms.^1,2,3,4^ The demographic characteristics of smokers who use flavored cigarettes, including menthol, is variable, but their use is typically more common among younger, newer smokers,^2,5,6,7,8,9^ and women.^6,8^ Cigarettes can also contain a capsule that is crushed or squeezed to release flavoring (usually menthol)^10^; capsule cigarettes are popular among younger smokers.^10,11,12^

US data suggest that the availability of menthol cigarettes can increase smoking initiation and slow decreases in smoking prevalence, thereby increasing smoking-related mortality.^13^ Among adult smokers, menthol cigarette smoking has been associated with greater dependence and reduced quit attempts and success.^9,14,15^ Among youth, initiation with menthol cigarettes facilitates progression to established use,^9^ and menthol smoking is associated with greater dependence.^9,16^ Banning menthol cigarettes could, therefore, accelerate decreases in smoking by reducing uptake and boosting cessation.

Bans on menthol cigarettes have been recommended by the World Health Organization and implemented in many jurisdictions.^17^ In 2009, Canada and the US banned flavors except menthol in cigarettes. In 2015, individual Canadian provinces became among the first jurisdictions globally to also ban menthol in cigarettes; over the next 2 years, 7 provinces also did so.^18^ In October 2017, Canada implemented a national ban.^19^ In May 2020, the European Union and England prohibited the sale of cigarettes with a characterizing flavor, including menthol.^20^ In the US, some states have implemented menthol cigarette bans, and the Food and Drug Administration has committed to a federal ban on menthol in cigarettes and cigars.^21^

Menthol cigarette markets also vary across countries. The US has among the highest market share (ie, proportion of sales in that market) of menthol cigarettes (36% in 2018),^22^ compared with 21% in England and 5% in Canada, before national bans.^19,22^ The US also has racial and ethnic differences in menthol smoking: 70% of youth and 80% of adult smokers who identify as Black smoke menthol, and almost one-half of menthol smokers are from minoritized racial and ethnic groups.^6^ This pattern is not seen among youth^23^ or adults^24^ in Canada. Little is known about racial and ethnic characteristics of menthol smokers in England.

The outcomes of menthol cigarette bans among adults in Canada were clear.^24,25,26,27,28^ After Ontario’s menthol ban, menthol cigarette sales neared zero, and total cigarette sales declined to a greater extent than in provinces without a ban.^26^ Menthol cigarette bans in Ontario^27,28^ and throughout Canada^24^ have also been longitudinally associated with higher rates of quit attempts and quit success among daily menthol smokers. In England, menthol cigarette smoking among adult smokers remained high 1 year after the ban, at 15.7%.^29^ In the US, banning menthol cigarettes and cigars is estimated to reduce overall smoking prevalence and smoking-related deaths.^30^ Evidence on the outcomes of menthol bans among youth outside of Canada is lacking.

The association of menthol cigarette bans with smokers’ characteristics warrants exploration. Because menthol smoking is more common among younger, newer smokers,^2,5,6,7,8,9^ female smokers,^6,8^ and, in the US, those who identify as Black,^6^ menthol bans may reduce smoking to a greater extent among these groups. However, menthol smokers who are more dependent may procure menthol cigarettes via illicit sources when they are banned, potentially leading to greater dependence among continuing menthol smokers.

This study aimed to evaluate the outcomes of menthol cigarette bans on menthol smoking among youth in England (where menthol cigarettes were banned in May 2020) and Canada (where menthol cigarettes were banned federally in October 2017), compared with the US (where menthol cigarettes are not banned federally), and to characterize menthol cigarette smokers in terms of demographics and consumption and dependence, across countries, and in England before and after the menthol ban. Our hypotheses were as follows: first, in England, menthol or capsule smoking among past 30-day smokers would decline after the ban, and this change would be greater than in the US and Canada. Second, the prevalence of menthol or capsule smoking among past 30-day smokers would be lower in Canada than the US and England. Third, the past 30-day menthol or capsule smokers in Canada would report greater cigarette consumption or dependence than those in the US and England.

## Methods

This survey study compared youth menthol smoking where menthol bans were implemented before the study (Canada), during the study (England), or not (US). Such natural experiments^31^ provide better evidence than observational studies because there is a natural control. Analyses were preregistered.^32^

### Data Source

Data were from the 2018 (August-September), 2019 (August-September), and 2020 (February-March, and August) waves of the online, repeat cross-sectional International Tobacco Control Youth Tobacco and Vaping Survey, conducted in Canada, England, and the US. Detailed methods, with information on data collection, response (using American Association for Public Opinion Research [AAPOR] reporting guideline), samples, weighting procedures, and quality checks are available online.^33,34,35^ Briefly, respondents aged 16 to 19 years were recruited through Nielsen consumer panels and received remuneration according to their panel’s incentive structure. This study received ethics clearance through the University of Waterloo Research Ethics Committee and the King’s College London Psychiatry, Nursing & Midwifery Research Ethics Subcommittee. Respondents were provided with study information and indicated consent to participate.

A total of 56 595 respondents completed the surveys, of whom 51 536 were in the full sample and 7067 were in the subsample of past 30-day smokers. Respondents were excluded if they failed data integrity checks (1908 respondents), had missing or incomplete data on variables required for calculating weights or determining smoking or vaping status (825 respondents), were recruited in a previous wave (2220 respondents), were an ineligible age (106 respondents), or, f‬or the main analyses, were not past 30-day smokers (44 414 respondents) or selected *refused* on outcomes (55 respondents). Full measure details are in the eAppendix in the Supplement.

### Outcomes

#### Menthol or Capsule as the Usual Brand or Variety of Cigarettes (Primary Outcome)

Past 30-day smokers were asked to select the brand and variety of cigarettes that they smoked most often from country-specific lists. Responses were classified as menthol or capsule vs all other responses.

#### Any Menthol or Flavor Capsule Cigarettes Use in the Past 30 Days (Secondary Outcome)

Past 30-day smokers were asked whether they had smoked any cigarettes that were flavored to taste like menthol or mint, and have a filter that was squeezed or crushed for flavor, in the past 30 days. Respondents who selected *Yes* to either were coded as menthol or capsule smokers vs all other responses. Although this measure asked about flavor (not menthol-specific) capsules, capsules in cigarettes are almost always menthol.^10,12^

### Exposure and Independent Variables

#### Exposure

Menthol cigarette bans were compared in countries where bans existed throughout the study (Canada), were implemented during the study (England), and were not implemented (US). Country (Canada, England, and US) and survey wave (2018, 2019, February 2020, and August 2020) were used as proxies for the policy.

#### Demographic Variables and Consumption and Dependence Indicators

Demographic variables included age group (16-17 vs 18-19 years), sex (male vs female), and race and ethnicity (any Black, White only, any other race [ie, any other response that was provided via a drop-down list that varied by country or was manually written in by participants as an open-ended response],^34^ multiracial, and do not know or refused to answer). Race and ethnicity were identified by the participants through the survey. Race and ethnicity were assessed in this study because race and ethnicity are variables of interest because of the racial disparities in menthol smoking seen in the US but not Canada and England. Consumption and dependence indicators included frequent smoking (≥20 of the past 30 days, otherwise), number of cigarettes per day (≤1, 2-5, >5, do not know or refused to answer), perceived addiction to cigarettes (a little or very, not at all, do not know or refused to answer), and urges to smoke (every day or most days, less often, or do not know or refused to answer).

### Statistical Analysis

#### Sample Weighting

Data analysis was performed from March 2021 to January 2022. Sample weighting details are available online.^33,34,35^ Briefly, cross-sectional poststratification sample weights were constructed for each country on the basis of population figures for sex-by-age-by-region (sex-by-age-by-region-by-race in the US), calibrated to wave 1 student status and school grades and past 30-day smoking trend in Canada and the US, and rescaled to each country’s sample size.

#### Association of Menthol Cigarette Bans With Youth Menthol Smoking

First, the proportion of past 30-day smokers who smoked menthol or capsule cigarettes was reported (7067 smokers). Second, logistic regression models adjusted for demographics were estimated, projecting menthol or capsule smoking from country and survey wave. Third, a country-by-survey wave interaction term was added to the regression models, and average adjusted probabilities were estimated from this model and contrasted at each time point within countries using the postestimation command *margins* in Stata statistical software version 17 (StataCorp).^36^ Past 30-day smoking among the full sample (51 536 respondents) was also reported (not preregistered).

#### Characteristics of Menthol and Capsule Smokers

Associations between demographics and menthol or capsule smoking were reported from the aforementioned logistic regression models adjusting for country and survey wave. Separate logistic regression models were then used to estimate menthol or capsule smoking from each consumption or dependence indicator, adjusting for country, survey wave, and demographics. Interaction terms between country and each demographic and consumption or dependence variable were added to the models in a subsequent step to examine whether associations differed across countries, and interactions were explored as described above.

In an additional step that was not preregistered, to examine changes in England after the ban, adjusted logistic regression models were used for England data only, estimating menthol or capsule smoking from survey wave and demographics (entered simultaneously) and each consumption or dependence indicator (entered separately into the adjusted models). As above, interaction terms between survey wave and each demographic and consumption or dependence variable were added to the models in a subsequent step.

#### Sensitivity Analyses

Analyses were stratified by past 30-day menthol (yes vs other) and capsule (yes vs other) smoking. Two-sided *P* < .05 was considered significant.

## Results

eTable 1 in the Supplement shows the sample characteristics. Our analysis focused on 7067 past 30-day smokers aged 16 to 19 years (4129 female; 5019 White).

### Association of Menthol Cigarette Bans With Youth Menthol Smoking

**Figure 1.**
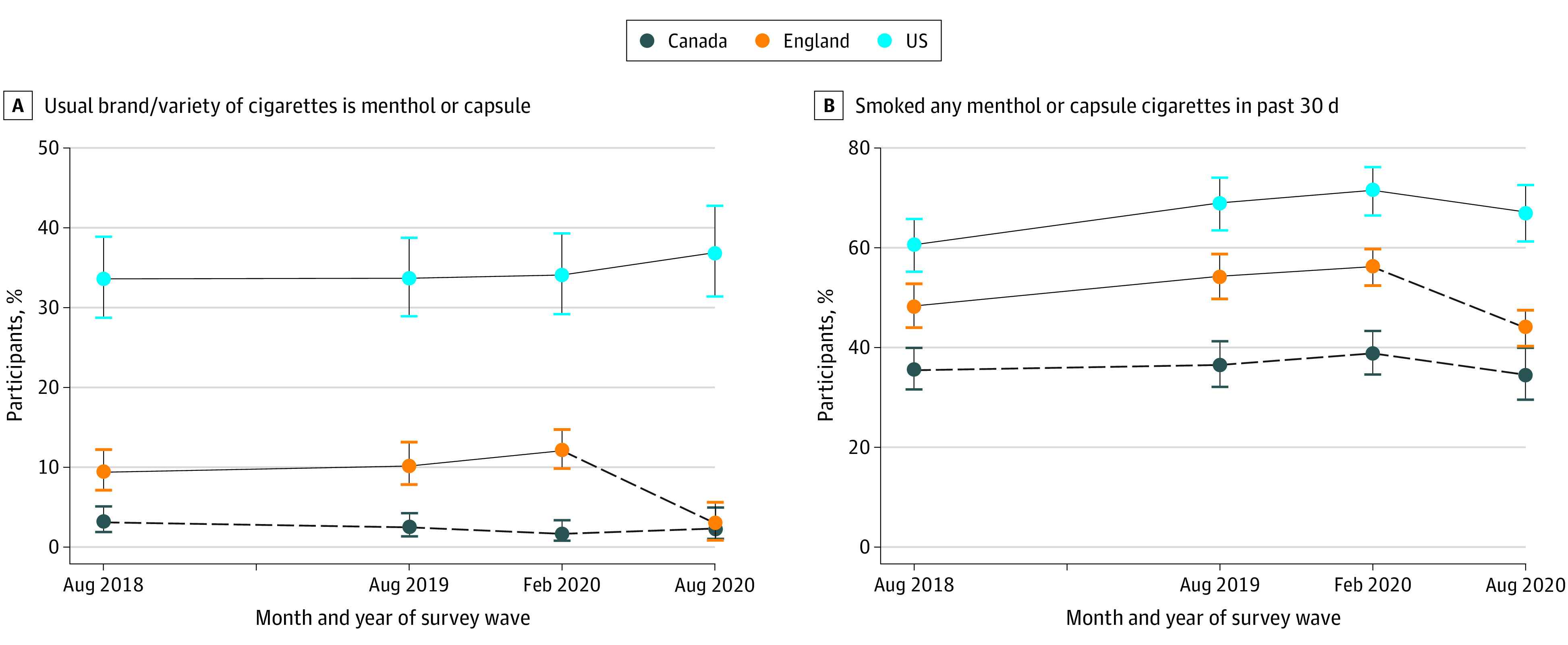
Proportion of Past 30-Day Smokers Who Smoked Menthol Cigarettes in England, Canada, and the US Graphs show data for 7067 smokers who reported a usual brand or variety of cigarettes that was menthol or capsule (A) and those who reported that they had smoked any menthol or capsule cigarettes in the past 30 days (B) between 2018 and 2020. Dashed lines indicate where a menthol cigarette ban was present.

Figure 1A shows the proportion of past 30-day smokers who reported a usual brand of cigarettes that was menthol or capsule. In England, the proportion of youth past 30-day smokers who reported usually smoking a menthol or capsule cigarette brand was stable before the menthol ban (2018 to February 2020, 9.4% vs 12.1%; adjusted odds ratio [AOR], 1.03; 95% CI, 0.99-1.06; *P* = .15) but decreased to 3.0% after the ban (August 2020 vs February 2020, 3.0% vs 12.1%; AOR, 1.07; 95% CI, 1.04-1.10; *P* < .001). There was also evidence that menthol or capsule smoking was lower in England after the ban, in August 2020, compared with all survey waves before the ban (eTable 2 in the Supplement). By contrast, menthol or capsule smoking was stable in the US (33.6% to 36.9%) and Canada (3.1% to 2.3%) throughout the study period (eTable 2 in the Supplement).

Across all survey waves, the proportion of youth past 30-day smokers who reported usually smoking a menthol or capsule cigarette brand was lower in Canada than in England (2.4% vs 8.9%; AOR, 4.07; 95% CI, 2.83-5.86; *P* < .001) and the US (2.4% vs 34.6%; AOR, 22.71; 95% CI, 15.92-32.39; *P* < .001). Prevalence was also higher in the US than England (AOR, 5.58; 95% CI, 4.63-6.72; *P* < .001). eTable 3 in the Supplement shows past 30-day smoking among the full sample.

### Characteristics of Menthol and Capsule Smokers

#### Demographics

eTable 4 and eTable 5 in the Supplement show the characteristics of menthol smokers. Table 1 shows the proportion of youth past 30-day smokers in each country who reported a usual cigarette brand that was menthol or capsule, by demographic characteristics. In Canada, there was little evidence for any demographic differences (Table 1). In England, menthol or capsule smoking was more common among female than male smokers (10.9% vs 7.2%; AOR, 1.04; 95% CI, 1.01-1.06; *P* = .002) (Table 1). In the US, menthol or capsule smoking was twice as common among past 30-day smokers who identified as Black (any) vs White only (60.6% vs 31.9%; AOR, 1.33; 95% CI, 1.23-1.44; *P* < .001) or who were frequent smokers, smoked more cigarettes per day, or had urges to smoke every or most days (Table 1).

**Table 1.** Percentages and Comparisons of Past 30-Day Smokers Who Reported a Usual Brand or Variety of Cigarettes That Was Menthol or Capsule or That They Had Smoked Any Menthol or Capsule Cigarettes in the Past 30 Days, by Demographics, in Canada, England, and the US^a^

Country and characteristic	Usual brand/variety of cigarettes is menthol or capsule (yes vs other)			Smoked any menthol or capsule cigarettes in the past 30 d (yes vs other)		
	Weighted %	AOR (95% CI)	*P* value	Weighted %	AOR (95% CI)	*P* value
Canada						
Age, y						
16-17	2.4	1 [Reference]	NA	35.5	1 [Reference]	NA
18-19	2.4	1.00 (0.98-1.02)	.96	37.1	1.00 (0.96-1.05)	.88
Sex						
Male	2.7	1 [Reference]	NA	38.7	1 [Reference]	NA
Female	1.9	0.99 (0.98-1.01)	.35	33.3	0.95 (0.91-0.99)	.02
Race and ethnicity						
Any Black	1.1	0.98 (0.97-1.00)	.10	49.3	1.22 (1.11-1.33)	<.001
White only	2.7	1 [Reference]	NA	29.6	1 [Reference]	NA
Any other or multiracial^b^	1.9	0.99 (0.98-1.01)	.27	44.9	1.17 (1.11-1.23)	<.001
Do not know or refused	3.7	1.09 (0.91-1.31)	.33	48.6	1.22 (1.01-1.47)	.04
England						
Age, y						
16-17	7.6	1 [Reference]	NA	45.0	1 [Reference]	NA
18-19	9.8	1.02 (1.00-1.04)	.11	55.3	1.10 (1.05-1.15)	<.001
Sex						
Male	7.2	1 [Reference]	NA	50.2	1 [Reference]	NA
Female	10.9	1.04 (1.01-1.06)	.002	51.8	1.01 (0.97-1.06)	.52
Race and ethnicity						
Any Black	6.2	0.97 (0.93-1.01)	.17	59.8	1.11 (1.01-1.21)	.03
White only	9.2	1 [Reference]	NA	49.4	1 [Reference]	NA
Any other or multiracial^b^	6.8	0.98 (0.94-1.01)	.19	57.3	1.08 (1.00-1.16)	.04
Do not know or refused	18.3	1.09 (0.91-1.31)^c^	.33	69.2	1.22 (1.01-1.47)^c^	.04
US						
Age, y						
16-17	34.9	1 [Reference]	NA	70.3	1 [Reference]	NA
18-19	34.3	0.99 (0.94-1.05)	.76	63.1	0.93 (0.88-0.98)	.006
Sex						
Male	33.6	1 [Reference]	NA	65.8	1 [Reference]	NA
Female	36.2	1.02 (0.97-1.07)	.33	67.9	1.02 (0.97-1.07)	.43
Race and ethnicity						
Any Black	60.6	1.33 (1.23-1.44)	<.001	77.0	1.12 (1.05-1.20)	.001
White only	31.9	1 [Reference]	NA	65.3	1 [Reference]	NA
Any other or multiracial^b^	32.7	1.01 (0.94-1.07)	.85	67.7	1.02 (0.96-1.09)	.51
Do not know or refused	41.0	1.10 (0.72-1.68)^c^	.67	48.2	0.85 (0.54-1.34)^c^	.48

Abbreviations: AOR, adjusted odds ratio; NA, not applicable.

^a^ Data are aggregated across survey waves (2018 to August 2020) for 7067 respondents. AORs, 95% CIs, and *P* values are derived from interactions from logistic regression models adjusting for country, survey wave, age, sex, and race and ethnicity.

^b^ Other race or ethnicity refers to any other response to race and ethnicity that did not fit within the categories of any Black or White only. Race and ethnicity were self-reported by respondents using a drop-down list that varied by country, or could also be self-reported by respondents as an open-ended response.

^c^ Treat estimate with caution (denominator is <30).

The AOR for being female and smoking menthol or capsule cigarettes was higher in England than in Canada (AOR, 2.12; 95% CI, 1.06-4.21; *P* = .03), and the AOR for identifying as Black (any) and smoking menthol or capsule cigarettes was higher in the US than in Canada (AOR, 8.12; 95% CI, 1.82-36.27; *P* = .006). There was little evidence for an interaction between country and age group (*F*_2,7065_ = 1.02; *P* = .36).

#### Consumption and Dependence Indicators

Table 2 shows the proportion of past 30-day smokers in each country who reported a usual brand that was menthol or capsule, by consumption and dependence indicators. In Canada, menthol or capsule smoking was more common among participants who reported being a little or very addicted to cigarettes than those who reported not being addicted (3.1% vs 1.3%; AOR, 1.02; 95% CI, 1.00-1.03; *P* = .01) (Table 2). In England, there was little evidence for any associations between cigarette consumption or dependence and menthol or capsule smoking (Table 2). In the US, menthol or capsule smoking was more common among those who smoked on at least 20 of the past 30 days vs otherwise (38.1% vs 32.7%; AOR, 1.07; 95% CI, 1.01-1.13; *P* = .03), who smoked 2 to 5 (37.9%) or more than 5 (38.5%) cigarettes per day than those who smoked 1 or fewer (29.8%) (2-5 vs 1 cigarettes per day, AOR, 1.09; 95% CI, 1.02-1.15; *P* = .006; >5 vs 1 cigarettes per day, AOR, 1.10; 95% CI, 1.03-1.18; *P* = .007), and who reported urges to smoke every or most days vs less often (38.0% vs 30.3%; AOR, 1.08; 95% CI, 1.02-1.14; *P* = .006) (Table 2).

**Table 2.** Percentages and Comparisons of Past 30-Day Smokers Who Reported a Usual Brand or Variety of Cigarettes That Was Menthol or Capsule or That They Had Smoked Any Menthol or Capsule Cigarettes in the Past 30 Days, by Consumption and Dependence Indicators, in Canada, England, and the US^a^

Country and indicator	Usual brand/variety of cigarettes is menthol or capsule (yes vs other)			Smoked any menthol or capsule cigarettes in the past 30 d (yes vs other)		
	Weighted %	AOR (95% CI)	*P* value	Weighted %	AOR (95% CI)	*P* value
Canada						
Frequent smoking						
Other	2.8	1 [Reference]	NA	36.6	1 [Reference]	NA
≥20 of past 30 d	1.7	0.99 (0.97-1.00)	.12	36.0	0.99 (0.95-1.04)	.82
Cigarettes per day, No.						
≤1	2.5	1 [Reference]	NA	35.8	1 [Reference]	NA
2-5	2.5	1.00 (0.98-1.02)	.91	39.6	1.04 (0.98-1.09)	.18
>5	2.2	1.00 (0.98-1.02)	.66	34.5	0.98 (0.93-1.04)	.57
Do not know or refused	0.0	NA^b^^,^^c^	NA	3.9	0.73 (0.69-0.78)^c^	<.001
Perceived addiction						
Not at all	1.3	1 [Reference]	NA	30.1	1 [Reference]	NA
A little/very	3.1	1.02 (1.00-1.03)	.01	40.4	1.10 (1.05-1.15)	<.001
Do not know or refused	0.0	NA^b^^,^^c^	NA	28.1	0.96 (0.80-1.14)^c^	.60
Urges to smoke						
Less often	2.0	1 [Reference]	NA	32.9	1 [Reference]	NA
Every or most days	3.0	1.01 (0.99-1.02)	.24	41.2	1.08 (1.04-1.13)	<.001
Do not know or refused	0.0	NA^b^^,^^c^	NA	14.5	0.83 (0.73-0.94)^c^	.003
England						
Frequent smoking						
Other	9.5	1 [Reference]	NA	48.9	1 [Reference]	NA
≥20 of past 30 d	7.6	0.98 (0.96-1.01)	.14	54.9	1.07 (1.03-1.12)	.002
Cigarettes per day, No.						
≤1	8.6	1 [Reference]	NA	45.1	1 [Reference]	NA
2-5	10.3	1.02 (0.99-1.04)	.25	55.4	1.11 (1.06-1.16)	<.001
>5	7.5	0.99 (0.96-1.02)	.51	56.0	1.13 (1.07-1.19)	<.001
Do not know or refused	2.0	0.94 (0.90-0.98)	.003	35.9	0.91 (0.77-1.07)	.25
Perceived addiction						
Not at all	9.5	1 [Reference]	NA	42.9	1 [Reference]	NA
A little/very	8.4	0.99 (0.97-1.01)	.44	57.0	1.15 (1.11-1.20)	<.001
Do not know or refused	3.9	0.95 (0.88-1.02)^c^	.16	33.7	0.92 (0.76-1.10)^c^	.36
Urges to smoke						
Less often	8.8	1 [Reference]	NA	47.2	1 [Reference]	NA
Every or most days	8.9	1.00 (0.98-1.03)	.86	56.0	1.10 (1.05-1.14)	<.001
Do not know or refused	11.5	1.03 (0.91-1.17)^c^	.66	34.8	0.90 (0.74-1.09)	.28
US						
Frequent smoking						
Other	32.7	1 [Reference]	NA	65.4	1 [Reference]	NA
≥20 of past 30 d	38.1	1.07 (1.01-1.13)	.03	68.8	1.04 (0.99-1.10)	.13
Cigarettes per day, No.						
≤1	29.8	1 [Reference]	NA	62.4	1 [Reference]	NA
2-5	37.9	1.09 (1.02-1.15)	.006	70.7	1.09 (1.02-1.16)	.007
>5	38.5	1.10 (1.03-1.18)	.007	69.3	1.08 (1.01-1.15)	.03
Do not know or refused	45.6	1.14 (0.88-1.49)^c^	.32	55.4	0.93 (0.69-1.25)^c^	.64
Perceived addiction						
Not at all	32.5	1 [Reference]	NA	57.5	1 [Reference]	NA
A little/very	35.1	1.03 (0.97-1.09)	.36	71.3	1.15 (1.09-1.22)	<.001
Do not know or refused	56.6	1.27 (1.03-1.58)^c^	.03	52.1	0.95 (0.75-1.21)^c^	.69
Urges to smoke						
Less often	30.3	1 [Reference]	NA	58.6	1 [Reference]	NA
Every or most days	38.0	1.08 (1.02-1.14)	.006	73.4	1.16 (1.10-1.22)	<.001
Do not know or refused	44.6	1.12 (0.85-1.48)^c^	.41	63.4	1.04 (0.78-1.38)^c^	.79

Abbreviations: AOR, adjusted odds ratio; NA, not applicable.

^a^ Data are aggregated across survey waves (2018 to August 2020) for 7067 respondents. AORs, 95% CIs, and P values are derived from interactions from logistic regression models adjusting for country, survey wave, age, sex, and race and ethnicity.

^b^ Estimate is unreportable because there were 0 respondents.

^c^ Treat estimate with caution (denominator is <30).

The AOR for smoking on at least 20 of the past 30 days and menthol or capsule smoking was higher in the US than in Canada (AOR, 2.33; 95% CI, 1.02-5.34; *P* = .046), but the AOR for addiction to smoking and menthol or capsule smoking was lower in England than in Canada (AOR, 0.37; 95% CI, 0.17-0.83; *P* = .02). There was little evidence for an interaction between country and cigarettes per day (*F*_5,7041_ = 1.75; *P* = .12) or country and urges to smoke (*F*_3,7036_ = 0.95; *P* = .42) when estimating menthol or capsule smoking.

#### Changes in Characteristics in England After the Ban

**Figure 2.**
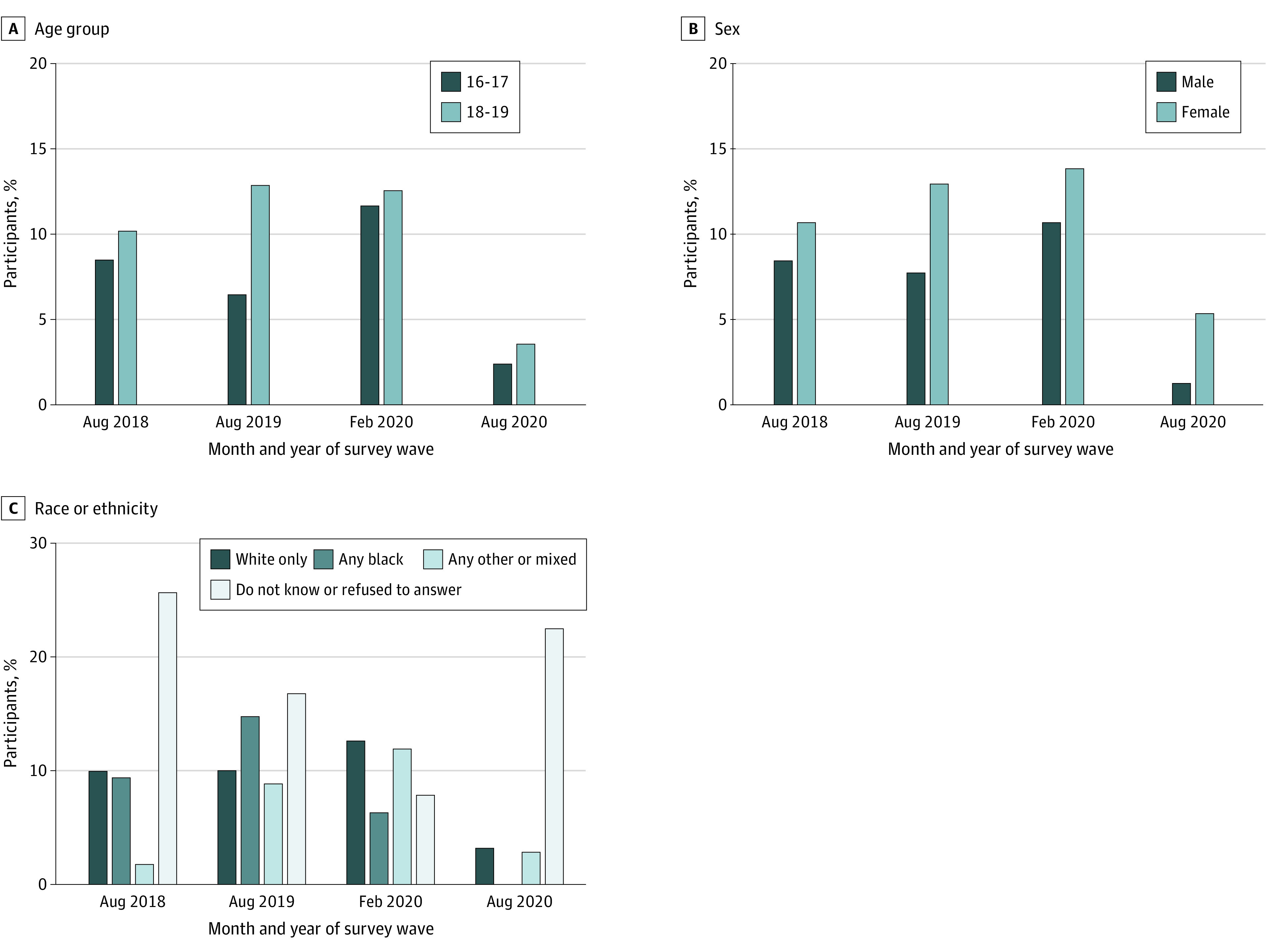
Proportion of 2843 Past 30-Day Smokers in England Who Reported a Usual Brand That Was Menthol or Capsule Over Time, by Demographic Characteristics

**Figure 3.**
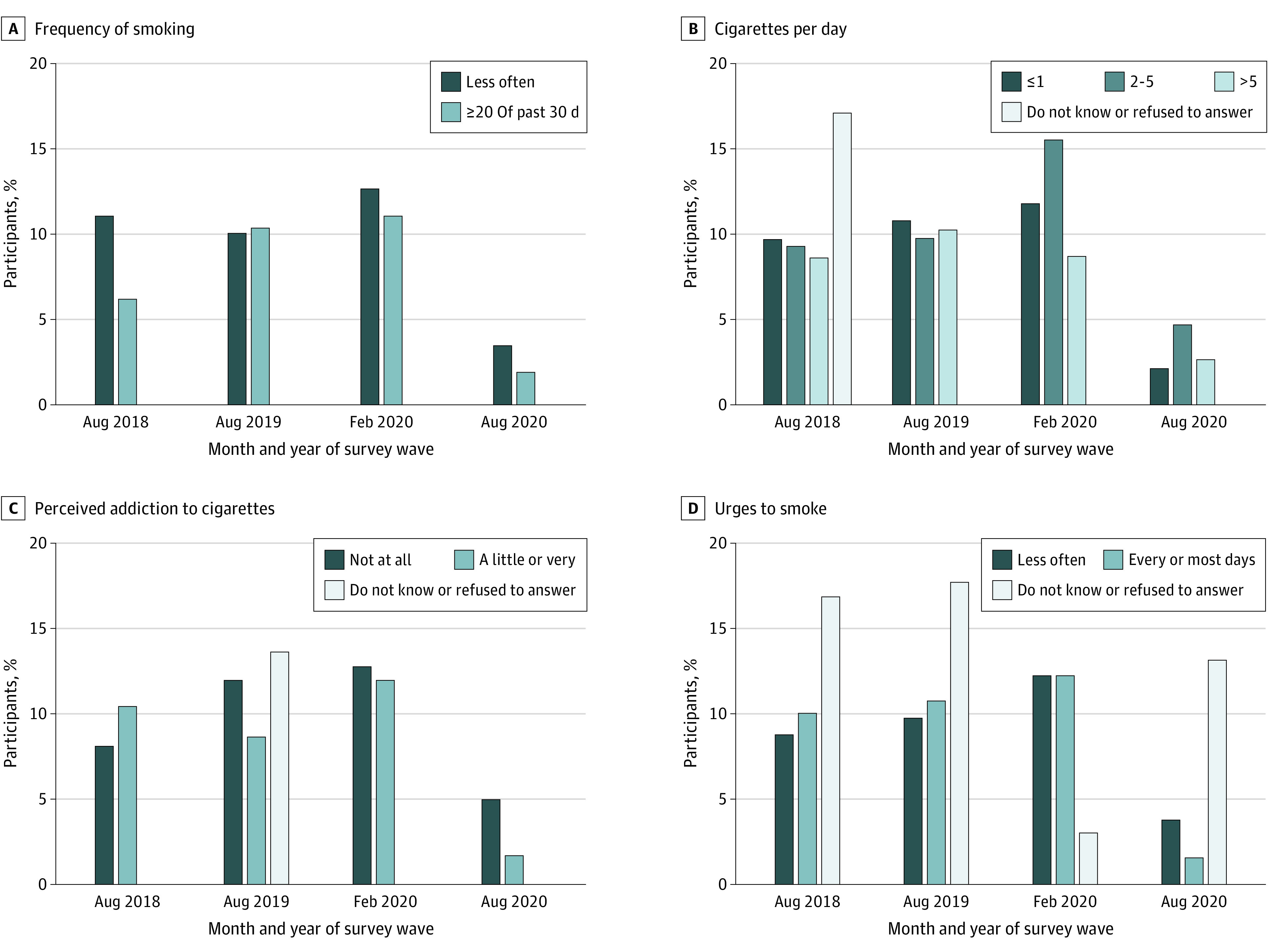
Proportion of 2843 Past 30-Day Smokers in England Who Reported a Usual Brand That Was Menthol or Capsule Over Time, by Consumption and Dependence Indicators

Figure 2 and Figure 3 show the proportion of past 30-day smokers in England who reported a usual brand that was menthol or capsule over time, by demographic characteristics and consumption and dependence indicators. There was little evidence that demographic differences in menthol or capsule smoking changed after the ban, in August 2020, compared with February 2020, by age group (18-19 vs 16-17 years, AOR, 1.35; 95% CI, 0.44-4.16; *P* = .60), sex (female vs male, AOR, 3.22; 95% CI, 0.93-11.14; *P* = .06), race (any other or multiracial vs White, AOR, 0.94; 95% CI, 0.15-5.94; *P* = .95; Black [any] vs White was unreportable because of the low sample size) (Figure 2). There was some evidence that the AOR for being a little or very addicted to cigarettes and smoking menthol or capsule cigarettes decreased after the ban, in August 2020, compared with February 2020 (AOR, 0.37; 95% CI, 0.41-0.97; *P* = .04) and also 2018 (AOR, 0.25; 95% CI, 0.09-0.71; *P* = .009) (Figure 3). There was little evidence for any other differences in menthol or capsule smoking by consumption and dependence indicators (Figure 3).

### Secondary Outcome: Smoked Any Menthol or Flavor Capsule Cigarettes in the Past 30 Days

Trends and country differences (Figure 1B and eTable 6 in the Supplement) and changes in characteristics of menthol or capsule smokers in England after the ban (eFigure 1 and 2 in the Supplement) were similar when using the secondary outcome of smoking any menthol or capsule cigarettes in the past 30 days, although prevalence was higher than the usual brand measure (44%-56% in England, 35%-39% in Canada, and 60%-72% in the US). However, there were several differences in the associations between demographics and consumption and dependence indicators and menthol or capsule smoking when using the secondary outcome (Table 1 and Table 2). Briefly, any menthol or capsule smoking in the past 30 days was less common among female smokers in Canada, more common among respondents who identified as Black in all 3 countries, and any other race or multiracial in Canada and England, and more common in England but less common in the US among smokers aged 18 to 19 years (Table 1). Any menthol or capsule smoking in the past 30 days was also associated with a greater number of consumption and dependence indicators in all 3 countries than the usual brand measure (Table 2).

### Sensitivity Analyses

Trends were similar when stratified by menthol (eTables 7-12 and eFigures 3-6 in the Supplement) and capsule (eTables 13-18 and eFigures 7-10 in the Supplement) smoking; in England, the proportion of youth smokers who reported usually smoking a menthol cigarette brand decreased from 4.0% in February 2020 to 0.3% in August 2020 (AOR, 1.04; 95% CI, 1.02-1.06; *P* < .001), whereas the proportion who reported usually smoking a capsule cigarette brand decreased from 8.1% in February 2020 to 2.7% in August 2020 (AOR, 1.02; 95% CI, 1.00-1.05; *P* = .03).

There were differences in between-country patterns for capsule smoking: unlike the overall and menthol findings, there was little evidence that capsule smoking differed between England and the US (eTable 18 in the Supplement). Furthermore, in England, a larger proportion of youth reported usually smoking capsule vs menthol cigarette brands, whereas the inverse was seen in Canada and the US.

## Discussion

To our knowledge, this survey study is the first to evaluate the outcomes of menthol cigarette bans among youth outside of Canada.^24,25,26,27,28^ Our findings supported 2 of 3 hypotheses. First, consistent with findings among adults in Canada,^24,25,26,27^ menthol (including capsule) cigarette smoking among past 30-day smokers declined in England after the ban, and this decline was greater than in the US and Canada, where menthol smoking remained stable. Second, consistent with market share estimates^19,22^ and given that menthol cigarettes have been banned since 2017 in Canada, menthol smoking among past 30-day smokers was lower in Canada than the US and England. Overall, findings show a clear association of menthol cigarette bans with reduced menthol smoking among youth smokers.

There was little evidence to support the third hypothesis that menthol smokers in Canada would report greater cigarette consumption or dependence than those in the US and England. By contrast, dependence was greater among menthol smokers in the US, and there was little evidence for country differences in consumption. Furthermore, in England, the association between perceived addiction to cigarettes and menthol smoking decreased after the ban. The hypothesis for Canada was based on the idea that more dependent or heavier smokers would continue to seek out menthol cigarettes when they are banned; however, this did not appear to be the case among youth.

This study also examined the demographics of menthol smokers, including changes in England after the ban. Consistent with prior research,^6,23^ in the US menthol smoking was twice as common among youth who identified as Black than White, but no racial or ethnic differences were observed in England or Canada. Also consistent with prior research in Europe,^8^ menthol smoking was more common among female smokers in England, but not the US or Canada. Despite this, in England, menthol smoking decreased to a similar extent after the ban among all youth, regardless of sex or race and ethnicity.

Our primary outcome was self-report of a usual brand or variety of cigarettes, selected from a country-specific list and coded as menthol or capsule. Brand loyalty is high among regular smokers,^37^ so this measure was used to reliably determine menthol cigarette smoking according to the markets in each country. We also assessed a secondary self-reported outcome of smoking any menthol cigarettes in the past 30 days, consistent with prior research.^23,25,27,28^ Prevalence of reporting any menthol smoking was markedly higher than reporting a usual brand or variety of cigarettes that is menthol; however, key findings were similar across both measures, demonstrating robustness.

The reasons for high prevalence of any menthol smoking in the past 30 days, including after menthol ban implementation in England (44%) and Canada (35%-39%), are unclear. Occasional menthol smoking via sharing or socially sourcing cigarettes may account for the higher prevalence of any use in contrast to the usual brand measure, but would do little to explain why prevalence remains high after the ban. Other potential explanations include use of menthol accessories to add menthol flavor to regular cigarettes, illicit sourcing, low compliance, and misreporting. Menthol accessories (eg, flavor cards, filter tips, sprays) have been marketed in England and Canada after bans and are popular among adult smokers,^29,38,39,40^ but would not be captured by the usual brand measure. Illicit sourcing and lack of compliance are also valid concerns. In Canada, provincial menthol bans have not appeared to increase illicit purchasing^28,41^ and manufacturer compliance has been high^42,43^; however, there is little research outside of Canada or among youth. Finally, youth may be misreporting other, noncigarette menthol nicotine products. For example, cigarillo sales have grown since the European Union menthol ban was announced,^39^ and menthol is a popular e-cigarette flavor.^44,45^ Future research should monitor menthol products and accessories used by youth, to further understand the broader impact of menthol bans.

We did not formally examine whether menthol cigarette bans were associated with reduced youth smoking overall; in line with conceptual models for evaluating tobacco control policies,^46^ we focused on the most specific outcome expected of the menthol cigarette ban—menthol cigarette smoking among smokers—rather than overall smoking prevalence, which may be confounded by other policy initiatives (eg, Tobacco 21 or tax increases) and COVID-19. Findings from Canada are mixed regarding substitution after provincial menthol cigarette bans, with one study finding that overall cigarette sales decreased,^26^ another finding that adult menthol smokers commonly switched to ban-exempt products (eg, flavored cigars),^47^ and 2 finding that most smokers substituted menthol with nonmenthol cigarettes.^24,48^ In our study, past 30-day smoking among youth in England increased shortly before the menthol cigarette ban, followed by a decrease immediately after the ban; as such, smoking prevalence remained relatively stable over the length of the study period. National surveys in England provide mixed findings over this period, with some finding that smoking prevalence among young people decreased between 2018 and 2020^49^ and others finding an increase.^50^ This variability may be due to COVID-19 restrictions, which affected smoking behaviors,^51,52,53^ and precludes attribution of changes in smoking prevalence to the menthol ban. Moreover, the impact of flavor restrictions on smoking initiation among young people is likely to occur gradually over the long term as youth age into the smoking initiation period without the inducement of flavored cigarettes. Examining substitution of menthol with nonmenthol cigarettes following national bans is, therefore, important for future research.

### Limitations and Strengths

This study has limitations that should be considered. First, the data for Canada and the US were weighted to reflect national smoking trends among youth, whereas data for England were not because of a lack of national smoking estimates among youth aged 16 to 19 years. However, prevalence estimates were similar to national benchmark surveys.^33,34,35^ Second, as mentioned already, the August 2020 wave occurred during COVID-19. Third, unmeasured confounding cannot be ruled out, although we selected variables that were most specific to the policy being evaluated, adjusted for demographics, and weighted the data to the populations from which the samples were derived.

Strengths of the study include the convergence of key findings across 2 measures of menthol smoking and large sample. Furthermore, we used a population-based survey among youth aged 16 to 19 years in England, Canada, and the US, enhancing the generalizability of the findings to these groups.

## Conclusions

The proportion of youth smokers who smoke menthol (including capsule) cigarettes decreased substantially following the menthol ban in England. This impact was similar among youth aged 16 to 17 and 18 to 19 years and by sex and racial and ethnic group. Perceived addiction among menthol smokers was also lower where menthol cigarettes were banned.

## Notes

Supplement. **eAppendix.** Measures **eTable 1. **Past 30-Day Cigarette Smoking by Survey Wave and Country Among the Full Sample (N=51,536) **eTable 2.** Sample Characteristics of Past 30-Day Smokers, by Survey Wave and Country (N=7,067) **eTable 3.** Number and Proportion of Past 30-Day Smokers Who Reported a Usual Brand/Variety of Cigarettes That Was Menthol or Capsule by Demographics, Frequent Smoking, Cigarettes Smoked per Day, Perceived Addiction, and Urges to Smoke, Split by Country and Survey Wave **eTable 4.** Number and Proportion of Past 30-Day Smokers Who Report Smoking Any Menthol or Capsule Cigarettes in the Past 30 Days by Demographics, Frequent Smoking, Cigarettes Smoked per Day, Perceived Addiction, and Urges to Smoke, Split by Country and Survey Wave **eTable 5.** Contrasts Within Countries for the Percentage of Past 30-Day Smokers Who Reported a) a Usual Brand/Variety of Cigarettes That Was Menthol or Capsule, b) That They Had Smoked Any Menthol or Capsule Cigarettes in the Past 30 Days (N=7,067) **eTable 6.** Adjusted Logistic Regression Models Predicting Menthol or Capsule Cigarette Smoking From Survey Wave, Country, and Demographic Covariates **eFigure 1.** Percentage of Past 30-Day Smokers Who Reported That Had Smoked Any Menthol or Capsule Cigarettes in the Past 30 Days by Demographic Characteristics in England Only at Each Survey Wave (N=2,843) **eFigure 2.** Percentage of Past 30-Day Smokers Who Reported That Had Smoked Any Menthol or Capsule Cigarettes in the Past 30 Days by Consumption/Dependence Indicators in England Only at Each Survey Wave (N=2,843) **eTable 7.** Number and Proportion of Past 30-Day Smokers Who Reported a Usual Brand/Variety of Cigarettes That Was Menthol Only (Not Capsule) by Demographics, Frequent Smoking, Cigarettes Smoked per Day, Perceived Addiction, and Urges to Smoke, Split by Country and Survey Wave **eTable 8.** Number and Proportion of Past 30-Day Smokers Who Report Smoking Any Menthol Only (Not Capsule) Cigarettes in the Past 30 Days by Demographics, Frequent Smoking, Cigarettes Smoked per Day, Perceived Addiction, and Urges to Smoke, Split by Country and Survey Wave **eTable 9.** Contrasts Within Countries for the Percentage of Past 30-Day Smokers Who Smoked Menthol Only (Not Capsule) Cigarettes by Survey Wave (N=7,067) **eTable 10.** Contrasts Within Countries for the Percentage of Past 30-Day Smokers Who Smoked Menthol Only (Not Capsule) Cigarettes by Demographics (N=7,067) **eTable 11.** Contrasts Within Countries for the Percentage of Past 30-Day Smokers Who Smoked Menthol Only (Not Capsule) Cigarettes by Consumption/Dependence Indicators (N=7,067) **eTable 12.** Adjusted Logistic Regression Models Predicting Menthol Only (Not Capsule) Cigarette Smoking From Survey Wave, Country, and Demographic Covariates **eFigure 3.** Percentage of Past 30-Day Smokers Who Reported a Usual Brand/Variety of Cigarettes That Was Menthol Only (Not Capsule) by Demographic Characteristics in England at Each Wave (N=2,843) **eFigure 4.** Percentage of Past 30-Day Smokers Who Reported That They Had Smoked Any Menthol Only (Not Capsule) Cigarettes by Demographic Characteristics in England at Each Wave (N=2,843) **eFigure 5.** Percentage of Past 30-Day Smokers Who Reported a Usual Brand/Variety of Cigarettes That Was Menthol Only (Not Capsule) by Consumption/Dependence Indicators in England at Each Wave (N=2,843) **eFigure 6.** Percentage of Past 30-Day Smokers Who Reported That They Had Smoked Any Menthol Only (Not Capsule) Cigarettes in the Past 30 Days by Consumption/Dependence Indicators in England at Each Wave (N=2,843) **eTable 13.** Number and Proportion of Past 30-Day Smokers Who Reported a Usual Brand/Variety of Cigarettes That Was Capsule (Not Menthol Only) by Demographics, Frequent Smoking, Cigarettes Smoked per Day, Perceived Addiction, and Urges to Smoke, Split by Country and Survey Wave **eTable 14.** Number and Proportion of Past 30-Day Smokers Who Report Smoking Any Capsule (Not Menthol Only) Cigarettes in the Past 30 Days by Demographics, Frequent Smoking, Cigarettes Smoked per Day, Perceived Addiction, and Urges to Smoke, Split by Country and Survey Wave **eTable 15.** Contrasts Within Countries for the Percentage of Past 30-Day Smokers Who Smoked Capsule (Not Menthol Only) Cigarettes by Survey Wave (N=7,067) **eTable 16.** Contrasts Within Countries for the Percentage of Past 30-Day Smokers Who Smoked Capsule (Not Menthol Only) Cigarettes by Demographics (N=7,067) **eTable 17.** Contrasts Within Countries for the Percentage of Past 30-Day Smokers Who Smoked Capsule (Not Menthol Only) Cigarettes by Consumption/Dependence Indicators (N=7,067) **eTable 18.** Adjusted Logistic Regression Models Predicting Capsule (Not Menthol Only) Cigarette Smoking From Survey Wave, Country, and Demographic Covariates **eFigure 7.** Percentage of Past 30-Day Smokers Who Reported a Usual Brand/Variety of Cigarettes That Was Capsule (Not Menthol Only) Cigarettes by Demographic Characteristics in England at Each Survey Wave (N=2,843) **eFigure 8.** Percentage of Past 30-Day Smokers Who Reported That They Had Smoked Any Capsule (Not Menthol Only) Cigarettes in the Past 30 Days by Demographic Characteristics in England at Each Survey Wave (N=2,843) **eFigure 9.** Percentage of Past 30-Day Smokers Who Reported a Usual Brand/Variety of Cigarettes That Was Capsule (Not Menthol Only) by Consumption/Dependence Indicators in England at Each Survey Wave (N=2,843) **eFigure 10.** Percentage of Past 30-Day Smokers Who Reported That They Had Smoked Any Capsule (Not Menthol Only) Cigarettes in the Past 30 Days by Consumption/Dependence Indicators in England at Each Survey Wave (N=2,843)
