# Supplementary material for: Evaluating the Outcomes of the Menthol Cigarette Ban in England by Comparing Menthol Cigarette Smoking Among Youth in England, Canada, and the US, 2018-2020
Source: JAMA Netw Open. 2022 May 3;5(5):e2210029. doi: 10.1001/jamanetworkopen.2022.10029 (PMC9066289; doi:10.1001/jamanetworkopen.2022.10029)
Supplement: Supplement. — eAppendix. Measures eTable 1. Past 30-Day Cigarette Smoking by Survey Wave and Country Among the Full Sample (N=51,536) eTable 2. Sample Characteristics of Past 30-Day Smokers, by Survey Wave and Country (N=7,067) eTable 3. Number and Proportion of Past 30-Day Smokers Who Reported a Usual Brand/Variety of Cigarettes That Was Menthol or Capsule by Demographics, Frequent Smoking, Cigarettes Smoked per Day, Perceived Addiction, and Urges to Smoke, Split by Country and Survey Wave eTable 4. Number and Proportion of Past 30-Day Smokers Who Report Smoking Any Menthol or Capsule Cigarettes in the Past 30 Days by Demographics, Frequent Smoking, Cigarettes Smoked per Day, Perceived Addiction, and Urges to Smoke, Split by Country and Survey Wave eTable 5. Contrasts Within Countries for the Percentage of Past 30-Day Smokers Who Reported a) a Usual Brand/Variety of Cigarettes That Was Menthol or Capsule, b) That They Had Smoked Any Menthol or Capsule Cigarettes in the Past 30 Days (N=7,067) eTable 6. Adjusted Logistic Regression Models Predicting Menthol or Capsule Cigarette Smoking From Survey Wave, Country, and Demographic Covariates eFigure 1. Percentage of Past 30-Day Smokers Who Reported That Had Smoked Any Menthol or Capsule Cigarettes in the Past 30 Days by Demographic Characteristics in England Only at Each Survey Wave (N=2,843) eFigure 2. Percentage of Past 30-Day Smokers Who Reported That Had Smoked Any Menthol or Capsule Cigarettes in the Past 30 Days by Consumption/Dependence Indicators in England Only at Each Survey Wave (N=2,843) eTable 7. Number and Proportion of Past 30-Day Smokers Who Reported a Usual Brand/Variety of Cigarettes That Was Menthol Only (Not Capsule) by Demographics, Frequent Smoking, Cigarettes Smoked per Day, Perceived Addiction, and Urges to Smoke, Split by Country and Survey Wave eTable 8. Number and Proportion of Past 30-Day Smokers Who Report Smoking Any Menthol Only (Not Capsule) Cigarettes in the Past 30 Days by Demographics, Frequent Smok [file jamanetwopen-e2210029-s001.pdf]

Supplemental Online Content

East KA, Reid JL, Burkhalter R, et al. Evaluating the outcomes of the menthol cigarette ban in England by comparing menthol cigarette smoking among youth in England, Canada, and the US, 2018-2020. *JAMA Netw Open*. 2022;5(5):e2210029. doi:10.1001/jamanetworkopen.2022.10029

eAppendix. Measures

eTable 1. Past 30-Day Cigarette Smoking by Survey Wave and Country Among the Full Sample (N=51,536)

eTable 2. Sample Characteristics of Past 30-Day Smokers, by Survey Wave and Country (N=7,067)

eTable 3. Number and Proportion of Past 30-Day Smokers Who Reported a Usual Brand/Variety of Cigarettes That Was Menthol or Capsule by Demographics, Frequent Smoking, Cigarettes Smoked per Day, Perceived Addiction, and Urges to Smoke, Split by Country and Survey Wave

eTable 4. Number and Proportion of Past 30-Day Smokers Who Report Smoking Any Menthol or Capsule Cigarettes in the Past 30 Days by Demographics, Frequent Smoking, Cigarettes Smoked per Day, Perceived Addiction, and Urges to Smoke, Split by Country and Survey Wave

eTable 5. Contrasts Within Countries for the Percentage of Past 30-Day Smokers Who Reported a) a Usual Brand/Variety of Cigarettes That Was Menthol or Capsule, b) That They Had Smoked Any Menthol or Capsule Cigarettes in the Past 30 Days (N=7,067)

eTable 6. Adjusted Logistic Regression Models Predicting Menthol or Capsule Cigarette Smoking From Survey Wave, Country, and Demographic Covariates

eFigure 1. Percentage of Past 30-Day Smokers Who Reported That Had Smoked Any Menthol or Capsule Cigarettes in the Past 30 Days by Demographic Characteristics in England Only at Each Survey Wave (N=2,843)

eFigure 2. Percentage of Past 30-Day Smokers Who Reported That Had Smoked Any Menthol or Capsule Cigarettes in the Past 30 Days by Consumption/Dependence Indicators in England Only at Each Survey Wave (N=2,843)

eTable 7. Number and Proportion of Past 30-Day Smokers Who Reported a Usual Brand/Variety of Cigarettes That Was Menthol Only (Not Capsule) by Demographics, Frequent Smoking, Cigarettes Smoked per Day, Perceived Addiction, and Urges to Smoke, Split by Country and Survey Wave

© 2022 East KA et al. *JAMA Network Open.*

eTable 8. Number and Proportion of Past 30-Day Smokers Who Report Smoking Any Menthol Only (Not Capsule) Cigarettes in the Past 30 Days by Demographics, Frequent Smoking, Cigarettes Smoked per Day, Perceived Addiction, and Urges to Smoke, Split by Country and Survey Wave

eTable 9. Contrasts Within Countries for the Percentage of Past 30-Day Smokers Who Smoked Menthol Only (Not Capsule) Cigarettes by Survey Wave (N=7,067)

eTable 10. Contrasts Within Countries for the Percentage of Past 30-Day Smokers Who Smoked Menthol Only (Not Capsule) Cigarettes by Demographics (N=7,067)

eTable 11. Contrasts Within Countries for the Percentage of Past 30-Day Smokers Who Smoked Menthol Only (Not Capsule) Cigarettes by Consumption/Dependence Indicators (N=7,067)

eTable 12. Adjusted Logistic Regression Models Predicting Menthol Only (Not Capsule) Cigarette Smoking From Survey Wave, Country, and Demographic Covariates

eFigure 3. Percentage of Past 30-Day Smokers Who Reported a Usual Brand/Variety of Cigarettes That Was Menthol Only (Not Capsule) by Demographic Characteristics in England at Each Wave (N=2,843)

eFigure 4. Percentage of Past 30-Day Smokers Who Reported That They Had Smoked Any Menthol Only (Not Capsule) Cigarettes by Demographic Characteristics in England at Each Wave (N=2,843)

eFigure 5. Percentage of Past 30-Day Smokers Who Reported a Usual Brand/Variety of Cigarettes That Was Menthol Only (Not Capsule) by Consumption/Dependence Indicators in England at Each Wave (N=2,843)

eFigure 6. Percentage of Past 30-Day Smokers Who Reported That They Had Smoked Any Menthol Only (Not Capsule) Cigarettes in the Past 30 Days by Consumption/Dependence Indicators in England at Each Wave (N=2,843)

eTable 13. Number and Proportion of Past 30-Day Smokers Who Reported a Usual Brand/Variety of Cigarettes That Was Capsule (Not Menthol Only) by Demographics, Frequent Smoking, Cigarettes Smoked per Day, Perceived Addiction, and Urges to Smoke, Split by Country and Survey Wave

eTable 14. Number and Proportion of Past 30-Day Smokers Who Report Smoking Any Capsule (Not Menthol Only) Cigarettes in the Past 30 Days by Demographics, Frequent Smoking, Cigarettes Smoked per Day, Perceived Addiction, and Urges to Smoke, Split by Country and Survey Wave

eTable 15. Contrasts Within Countries for the Percentage of Past 30-Day Smokers Who Smoked Capsule (Not Menthol Only) Cigarettes by Survey Wave (N=7,067)

eTable 16. Contrasts Within Countries for the Percentage of Past 30-Day Smokers Who Smoked Capsule (Not Menthol Only) Cigarettes by Demographics (N=7,067)

© 2022 East KA et al. *JAMA Network Open.*

eTable 17. Contrasts Within Countries for the Percentage of Past 30-Day Smokers Who Smoked Capsule (Not Menthol Only) Cigarettes by Consumption/Dependence Indicators (N=7,067)

eTable 18. Adjusted Logistic Regression Models Predicting Capsule (Not Menthol Only) Cigarette Smoking From Survey Wave, Country, and Demographic Covariates

eFigure 7. Percentage of Past 30-Day Smokers Who Reported a Usual Brand/Variety of Cigarettes That Was Capsule (Not Menthol Only) Cigarettes by Demographic Characteristics in England at Each Survey Wave (N=2,843)

eFigure 8. Percentage of Past 30-Day Smokers Who Reported That They Had Smoked Any Capsule (Not Menthol Only) Cigarettes in the Past 30 Days by Demographic Characteristics in England at Each Survey Wave (N=2,843)

eFigure 9. Percentage of Past 30-Day Smokers Who Reported a Usual Brand/Variety of Cigarettes That Was Capsule (Not Menthol Only) by Consumption/Dependence Indicators in England at Each Survey Wave (N=2,843)

eFigure 10. Percentage of Past 30-Day Smokers Who Reported That They Had Smoked Any Capsule (Not Menthol Only) Cigarettes in the Past 30 Days by Consumption/Dependence Indicators in England at Each Survey Wave (N=2,843)

This supplemental material has been provided by the authors to give readers additional information about their work.

eAppendix. Measures

Outcomes

Usual brand/variety of cigarettes smoked is menthol or menthol capsule (primary outcome). Respondents who smoked in the past 30 days were first asked about the ***brand*** of cigarettes that they smoked: “What specific brand of cigarettes or roll-your-own (RYO) tobacco do you currently smoke most often?” followed by a country-specific list of brands, or response options ‘Unbranded cigarettes (“baggies”)’, ‘Other brand (please specify) ___’ (with an open-ended box for respondents to specify), ‘I don’t have a usual brand’, ‘Don’t know’, ‘Refused’. These same respondents were then also asked about the ***variety*** of cigarettes that they smoked: “What specific variety of cigarettes or roll-your-own tobacco do you currently smoke most often?” followed by a country- and brand-specific list of varieties, or response options ‘Other variety ___’ (with an open-ended box for respondents to specify), ‘I don’t have a usual variety’, ‘Don’t know’, ‘Refused’. The list of brands and varieties were specific to each country, based on a scan of retail outlets in each country and responses to previous survey waves.[32-34] All brands and varieties were manually coded as menthol, menthol capsule, or neither by two authors independently (KE and JLR; 91% agreement) and disagreements were resolved through discussion and/or further research where necessary. In the main analyses, responses were coded as menthol/capsule vs. not. Responses ‘I don’t have a usual brand/variety’ (n=435, 6.3%) and ‘Don’t know’ (n=206, 2.7%) were coded as not menthol/capsule. Respondents who selected ‘Refused’ were coded as missing and excluded from the analytic sample.

Smoked any menthol or flavour capsule cigarettes in the past 30 days (secondary outcome). Respondents who smoked in the past 30 days were asked, a) “In the past 30 days, were any of the cigarettes you smoked flavoured to taste like menthol or mint? Yes; No; Don’t know; Refused”; and, b) “In the past 30 days, did any of the cigarettes you smoked have a filter that you squeeze or crush for flavour? Yes; No; Don’t know; Refused”. Respondents who selected ‘Yes’ to a) or b) were coded as menthol or flavour capsule smokers. All other respondents were coded as ‘other’. Respondents who selected ‘Refused’ on either a) or b) were coded as missing. While this measure asked about flavour (not menthol-specific) capsules, capsules in cigarettes almost always release a menthol flavour.[11]

© 2022 East KA et al. *JAMA Network Open.*

Consumption/dependence indicators

Frequent smoking. Respondents who smoked were asked, “In the past 30 days, on how many days did you smoke cigarettes?” followed by a box in which they could enter a number within the range 0-30, or “Don’t know” or “Refused”. Respondents who answered 20 or greater were coded as smoking on at least 20 of the past 30 days vs. other.[1]

Cigarettes smoked per day. Respondents who smoked were asked, “In the past 30 days, on the days you smoked, how many cigarettes did you smoke per day? Less than 1 cigarette per day; 1 cigarette per day; 2 to 5 cigarettes per day; 6 to 10 cigarettes per day; 11 to 20 cigarettes per day; More than 20 cigarettes per day; Don’t know; Refused”. Responses were coded as ≤1 cigarette per day, 2-5 cigarettes per day, and >5 cigarettes per day. ‘Don’t know’ and ‘Refused’ responses were combined and coded as ‘Don’t know/refused’.

Perceived addiction to cigarettes. Respondents who smoked were asked, “Do you consider yourself addicted to cigarettes? Not at all; Yes, a little addicted; Yes, very addicted; Don’t know; Refused”. Respondents who answered ‘Yes, very addicted’ or ‘Yes, a little addicted’ were coded as a little or very addicted, vs. ‘Not at all’. ‘Don’t know’ and ‘Refused’ responses were combined and coded as ‘Don’t know/refused’.

Urges to smoke. Respondents who smoked were asked, “In the past 30 days, how often did you have a strong urge to smoke a cigarette? Several times a day; Every day or most days; At least once a week; Less than once a week; Never; Don’t know; Refused”. Respondents who answered ‘Several times a day’ or ‘Every day or most days’ were coded as having urges to smoke every day or most days, vs. less often (‘At least once a week’, ‘Less than once a week’ or ‘Never’). ‘Don’t know’ and ‘Refused’ responses were combined and coded as ‘Don’t know/refused’.

[1] In the pre-registration (https://osf.io/q2bmj) we specified that we would separate out ‘Don’t know’ and ‘Refused’ responses for Frequent Smoking. However, the survey was designed such that all past 30-day smokers were assigned a valid value (yes, no) in response to the Frequent Smoking item; this coding was therefore kept for consistency with other publications using these data.

© 2022 East KA et al. *JAMA Network Open.*

eTable 1. Past 30-Day Cigarette Smoking by Survey Wave and Country Among the Full Sample (N=51,536)

	CANADA	ENGLAND	US
Aug 2018	13.9%	16.7%	11.7%
Aug 2019	11.9%	14.8%	13.9%
Feb 2020	12.5%	19.6%	12.6%
Aug 2020	7.8%	15.1%	9.8%

Data are weighted based on population figures for sex-by-age-by-region (sex-by-age-by-region-by-race in the US), calibrated to Wave 1 student status and school grades. Note that these weights differ from those described in the manuscript and used in all other analyses, which also account for the past 30-day smoking trend in Canadas and the US.

© 2022 East KA et al. *JAMA Network Open.*

eTable 2. Sample Characteristics of Past 30-Day Smokers, by Survey Wave and Country (N=7,067)

	CANADA (n=2113)				ENGLAND (n=2843)				US (n=2111)			
	Aug 2018 (n=584)	Aug 2019 (n=557)	Feb 2020 (n=614)	Aug 2020 (n=358)	Aug 2018 (n=634)	Aug 2019 (n=588)	Feb 2020 (n=936)	Aug 2020 (n=685)	Aug 2018 (n=445)	Aug 2019 (n=548)	Feb 2020 (n=630)	Aug 2020 (n=488)
**AGE GROUP**												
16-17	49.2 (233)	38.6 (186)	42.2 (239)	48.2 (185)	45.5 (176)	41.3 (204)	42.2 (352)	42.3 (210)	48.0 (222)	45.6 (211)	48.9 (313)	50.3 (255)
18-19	50.8 (351)	61.4 (371)	57.8 (375)	51.8 (173)	54.5 (458)	58.7 (384)	57.8 (584)	57.7 (475)	52.0 (223)	54.4 (337)	51.1 (317)	49.7 (233)
**SEX**												
Male	60.5 (311)	57.3 (224)	53.4 (244)	54.5 (155)	57.0 (255)	52.6 (213)	52.9 (363)	56.4 (273)	61.3 (209)	60.1 (204)	61.7 (264)	67.5 (223)
Female	39.5 (273)	42.7 (333)	46.6 (370)	45.5 (203)	43.0 (379)	47.4 (375)	47.1 (573)	43.6 (412)	38.7 (236)	39.9 (344)	38.3 (366)	32.5 (265)
**RACE/ETHNICITY**												
White only	47.4 (281)	60.3 (327)	65.4 (398)	63.0 (229)	85.4 (531)	82.4 (486)	85.4 (791)	84.0 (572)	75.0 (297)	80.8 (362)	76.8 (421)	82.8 (324)
Any Black	7.4 (44)	8.0 (45)	6.5 (41)	6.1 (22)	4.1 (31)	4.0 (29)	5.1 (59)	6.7 (48)	10.0 (60)	8.4 (83)	9.1 (81)	7.6 (64)
Any other or mixed	29.9 (175)	29.0 (166)	26.1 (163)	29.3 (100)	9.3 (66)	12.3 (64)	8.9 (78)	8.9 (62)	14.5 (86)	10.7 (100)	13.8 (126)	9.5 (98)
Don't know/refused	15.2 (84)	2.7 (19)	2.0 (12)	1.6 (7)	1.1 (6)	1.3 (9)	0.6 (8)	0.4 (3)	0.5 (2)	0.1 (3)	0.4 (2)	0.1 (2)

Data are weighted % (unweighted n).

eTable 3. Number and Proportion of Past 30-Day Smokers Who Reported a Usual Brand/Variety of Cigarettes That Was Menthol or Capsule by Demographics, Frequent Smoking, Cigarettes Smoked per Day, Perceived Addiction, and Urges to Smoke, Split by Country and Survey Wave. Data are weighted % (unweighted n).

	CANADA				ENGLAND				US			
	Aug 2018 (N=584)	Aug 2019 (N=557)	Feb 2020 (N=614)	Aug 2020 (N=358)	Aug 2018 (N=634)	Aug 2019 (N=588)	Feb 2020 (N=936)	Aug 2020 (N=685)	Aug 2018 (N=445)	Aug 2019 (N=548)	Feb 2020 (N=630)	Aug 2020 (N=488)
**AGE GROUP**												
16-17	2.7 (6)	2.3 (5)	2.5 (5)	2.0 (3)	8.5 (15)	6.4 (12)	11.6 (41)	2.4 (5)	35.2 (76)	33.3 (74)	34.8 (114)	35.7 (96)
18-19	3.5 (10)	2.6 (9)	1.0 (3)	2.7 (4)	10.2 (48)	12.8 (54)	12.5 (76)	3.5 (21)	32.2 (73)	34.0 (134)	33.4 (119)	38.1 (84)
**SEX**												
Male	3.2 (9)	2.4 (6)	2.2 (5)	3.1 (4)	8.4 (22)	7.7 (16)	10.7 (39)	1.3 (4)	34.3 (76)	30.4 (70)	30.5 (84)	37.8 (82)
Female	2.9 (7)	2.5 (8)	1.1 (3)	1.4 (3)	10.7 (41)	12.9 (50)	13.8 (78)	5.3 (22)	32.5 (73)	38.5 (138)	39.9 (149)	35.1 (98)
**RACE/ETHNICITY**												
White only	2.8 (6)	3.3 (11)	2.1 (6)	2.7 (4)	10.0 (57)	10.1 (56)	12.5 (102)	3.2 (23)	30.7 (90)	31.3 (126)	29.1 (123)	36.2 (109)
Any black	0.0 (0)	1.8 (1)	2.5 (1)	0.0 (0)	9.4 (3)	14.8 (4)	6.3 (4)	0.0 (0)	65.3 (34)	51.3 (44)	68.6 (55)	52.2 (36)
Any other	3.3 (6)	1.2 (2)	0.5 (1)	2.2 (3)	1.8 (2)	8.9 (5)	11.9 (10)	2.9 (2)	26.5 (24)	37.9 (37)	39.0 (54)	30.8 (34)
Don't know/refused	5.3 (4)	0.0 (0)	0.0 (0)	0.0 (0)	25.7 (1)	16.8 (1)	7.9 (1)	22.5 (1)	46.1 (1)	26.3 (1)	30.9 (1)	60.9 (1)
**FREQUENT SMOKING**												
Other	3.7 (11)	2.9 (11)	2.1 (7)	2.4 (6)	11.1 (47)	10.1 (43)	12.7 (76)	3.5 (19)	30.2 (92)	32.8 (136)	33.5 (148)	35.3 (104)
≥20 of past 30 days	2.2 (5)	1.4 (3)	0.7 (1)	2.1 (1)	6.2 (16)	10.4 (23)	11.1 (41)	2.0 (7)	40.4 (57)	35.5 (72)	35.3 (85)	39.4 (76)
**CIGARETTES PER DAY**												
≤1	3.2 (7)	3.0 (8)	1.3 (3)	2.5 (4)	9.8 (26)	10.8 (26)	11.8 (51)	2.1 (6)	27.9 (55)	32.7 (83)	32.5 (96)	27.0 (65)
2 to 5	2.9 (5)	2.7 (4)	2.6 (4)	1.6 (2)	9.3 (25)	9.7 (24)	15.5 (44)	4.7 (16)	33.7 (51)	36.8 (74)	37.6 (89)	43.9 (65)
>5	3.2 (4)	1.2 (2)	1.0 (1)	3.0 (1)	8.6 (11)	10.3 (16)	8.7 (22)	2.7 (4)	42.6 (38)	31.8 (49)	32.3 (47)	43.5 (50)
Don't know/refused	0.0 (0)	0.0 (0)	0.0 (0)	0.0 (0)	17.1 (1)	0.0 (0)	0.0 (0)	0.0 (0)	82.8 (5)	21.7 (2)	18.1 (1)	0.0 (0)
**PERCEIVED ADDICTION**												
Not at all	3.4 (6)	1.7 (4)	0.0 (0)	0.0 (0)	8.1 (26)	12.0 (30)	12.8 (46)	5.0 (15)	35.0 (57)	29.2 (59)	29.8 (68)	34.6 (55)
A little/very	3.0 (10)	3.0 (10)	2.6 (8)	3.9 (7)	10.4 (37)	8.7 (35)	12.0 (71)	1.7 (11)	32.1 (88)	36.3 (147)	35.5 (159)	37.3 (121)
Don't know/refused	0.0 (0)	0.0 (0)	0.0 (0)	0.0 (0)	0.0 (0)	13.6 (1)	0.0 (0)	0.0 (0)	59.9 (4)	7.4 (2)	77.1 (6)	78.2 (4)
**URGES TO SMOKE**												
Less often	3.5 (8)	2.5 (8)	1.4 (4)	0.6 (1)	8.8 (36)	9.7 (36)	12.2 (58)	3.8 (17)	28.1 (58)	29.3 (80)	32.0 (98)	32.3 (68)
Every or most days	2.8 (8)	2.6 (6)	2.0 (4)	4.8 (6)	10 (26)	10.7 (29)	12.2 (58)	1.6 (8)	37.6 (87)	36.5 (127)	36.7 (134)	41.0 (112)
Don’t know/refused	0.0 (0)	0.0 (0)	0.0 (0)	0.0 (0)	16.8 (1)	17.7 (1)	3.0 (1)	13.1 (1)	76.0 (4)	49.9 (1)	6.4 (1)	0.0 (0)

© 2022 East KA et al. *JAMA Network Open.*

eTable 4. Number and Proportion of Past 30-Day Smokers Who Report Smoking Any Menthol or Capsule Cigarettes in the Past 30 Days by Demographics, Frequent Smoking, Cigarettes Smoked per Day, Perceived Addiction, and Urges to Smoke, Split by Country and Survey Wave. Data are weighted % (unweighted n).

	CANADA				ENGLAND				US			
	**Aug 2018 (N=584)**	**Aug 2019 (N=557)**	**Feb 2020 (N=614)**	**Aug 2020 (N=358)**	**Aug 2018 (N=634)**	**Aug 2019 (N=588)**	**Feb 2020 (N=936)**	**Aug 2020 (N=685)**	**Aug 2018 (N=445)**	**Aug 2019 (N=548)**	**Feb 2020 (N=630)**	**Aug 2020 (N=488)**
**AGE GROUP**												
16-17	31.9 (74)	38.1 (66)	36.7 (90)	36.0 (63)	43.7 (77)	50.2 (101)	47.4 (170)	39.3 (83)	66.2 (144)	70.3 (152)	72.9 (226)	72.7 (184)
18-19	39.1 (127)	35.5 (121)	40.3 (136)	33.1 (56)	52.2 (237)	57.2 (231)	62.4 (361)	47.4 (230)	55.4 (130)	67.8 (238)	70.2 (222)	61.6 (148)
**SEX**												
Male	39.1 (122)	40.3 (84)	39.2 (89)	36 (55)	48.8 (128)	49.9 (107)	55.3 (200)	45.5 (129)	59.8 (130)	67.4 (142)	69.0 (181)	68.5 (159)
Female	30.2 (79)	31.4 (103)	38.4 (137)	32.7 (64)	47.7 (186)	59.2 (225)	57 (331)	41.9 (184)	61.8 (144)	71.4 (248)	75.7 (267)	64.5 (173)
**RACE/ETHNICITY**												
White only	24.9 (68)	33.5 (99)	32.3 (121)	26.4 (60)	46.0 (252)	52.2 (268)	56 (445)	42.1 (250)	59.6 (178)	66.8 (250)	70.6 (298)	65.7 (211)
Any black	51.5 (21)	42.0 (21)	60.1 (24)	44.4 (9)	59.3 (19)	64.8 (18)	64.7 (38)	52.9 (27)	67.9 (41)	84.3 (66)	84 (63)	76.8 (50)
Any other	37.5 (66)	42.8 (64)	49.0 (76)	50.9 (49)	61.4 (39)	65.8 (42)	51.0 (43)	51.9 (33)	60.9 (54)	73.4 (72)	69.5 (86)	72.6 (70)
Don't know/refused	57.1 (46)	19.7 (3)	47.8 (5)	16.5 (1)	79.1 (4)	46.9 (4)	69.5 (5)	100.0 (3)	53.9 (1)	59.0 (2)	30.9 (1)	60.9 (1)
**FREQUENT SMOKING**												
Other	30.7 (104)	38.0 (132)	39.4 (160)	37.2 (92)	48.4 (209)	50.9 (209)	53.4 (322)	42.0 (198)	59.9 (183)	69.6 (259)	68.9 (287)	65.2 (206)
≥20 of past 30 days	42.4 (97)	33.1 (55)	37.6 (66)	27.1 (27)	48.3 (105)	61.7 (123)	61.1 (209)	48.0 (115)	61.9 (91)	67.5 (131)	77.4 (161)	70.3 (126)
**CIGARETTES PER DAY**												
≤1	33.4 (81)	37.8 (92)	37.1 (105)	34.3 (60)	41.8 (117)	45.2 (109)	51.5 (200)	40.2 (122)	54.7 (110)	72.4 (167)	65.5 (186)	60.8 (127)
2 to 5	38.8 (71)	37.3 (55)	45.0 (90)	36.3 (39)	54.8 (137)	56.6 (123)	59.9 (188)	49.0 (111)	63.4 (94)	67.6 (128)	77.9 (157)	74.6 (119)
>5	36.4 (49)	33.5 (39)	34.3 (31)	33.2 (20)	49.5 (58)	67.5 (96)	60.3 (137)	46.9 (78)	69.0 (65)	63.9 (92)	75.5 (103)	68.7 (84)
Don't know/refused	0.0 (0)	16.3 (1)	0.0 (0)	0.0 (0)	61.2 (2)	52.8 (4)	40.4 (6)	10.7 (2)	69.4 (5)	69.5 (3)	28.7 (2)	23.1 (2)
**PERCEIVED ADDICTION**												
Not at all	24.9 (50)	36.6 (72)	30.8 (58)	27.3 (36)	41.5 (139)	43.8 (116)	50.7 (175)	34.0 (105)	50.9 (87)	65.6 (110)	60.2 (127)	56.7 (93)
A little/very	42.5 (150)	37.4 (115)	43.0 (163)	38.3 (80)	54.1 (175)	62.9 (211)	60.3 (353)	51.0 (205)	65.9 (183)	71.6 (278)	76.9 (316)	72.0 (235)
Don't know/refused	7.8 (1)	0.0 (0)	39.3 (5)	79.3 (3)	0.0 (0)	53.8 (5)	21.4 (3)	33.0 (3)	52.3 (4)	6.8 (2)	70.0 (5)	78.2 (4)
**URGES TO SMOKE**												
Less often	27.7 (79)	34.4 (97)	35.3 (106)	33.7 (66)	45.0 (178)	49.2 (172)	52.9 (253)	40.7 (152)	47.7 (99)	69.7 (149)	61.9 (175)	61.0 (136)
Every or most days	43.9 (121)	39.8 (88)	44.0 (119)	36.3 (53)	52.1 (133)	62.9 (159)	60.0 (275)	49.1 (157)	72.1 (171)	68.1 (239)	81.2 (268)	72.7 (196)
Don’t know/refused	15.2 (1)	26.6 (2)	10.6 (1)	0.0 (0)	65.1 (3)	19.6 (1)	40.1 (3)	24.6 (4)	72.0 (4)	100.0 (2)	43.6 (5)	0.0 (0)

© 2022 East KA et al. *JAMA Network Open.*

eTable 5. Contrasts Within Countries for the Percentage of Past 30-Day Smokers Who Reported a) a Usual Brand/Variety of Cigarettes That Was Menthol or Capsule, b) That They Had Smoked Any Menthol or Capsule Cigarettes in the Past 30 Days (N=7,067).

	Usual brand/variety of cigarettes is menthol or capsule (yes vs. other)			Smoked any menthol or capsule cigarettes in the past 30 days (yes vs. other)		
	**%**	**AOR (95% CI)**	**p**	**%**	**AOR (95% CI)**	**p**
**ENGLAND**						
Aug 2020	3.0	REF		43.9	REF	
Feb 2020	12.1	**1.07 (1.04-1.10)**	**<.001**	56.1	1.05 (0.99-1.11)	.129
Aug 2019	10.2	**1.08 (1.04-1.11)**	**<.001**	54.3	**1.11 (1.04-1.18)**	**.001**
Aug 2018	9.4	**1.10 (1.07-1.13)**	**<.001**	48.3	**1.13 (1.07-1.20)**	**<.001**
**CANADA**						
Aug 2020	2.3	REF		34.5	REF	
Feb 2020	1.7	1.01 (0.98-1.03)	.664	38.8	0.99 (0.92-1.05)	.654
Aug 2019	2.5	1.00 (0.98-1.02)	.943	36.5	1.01 (0.95-1.08)	.703
Aug 2018	3.1	0.99 (0.97-1.02)	.539	35.6	1.04 (0.98-1.12)	.207
**US**						
Aug 2020	36.9	REF		67.2	REF	
Feb 2020	34.1	0.96 (0.89-1.04)	.307	71.5	0.93 (0.86-1.00)	.061
Aug 2019	33.7	0.96 (0.89-1.04)	.325	69.0	1.01 (0.94-1.10)	.708
Aug 2018	33.6	0.97 (0.90-1.04)	.394	60.6	1.04 (0.96-1.12)	.322

Data are weighted. Contrasts (AOR, 95% CI, p) are derived from interactions from logistic regression models adjusted for age group, sex, and race/ethnicity.

© 2022 East KA et al. *JAMA Network Open.*

eTable 6. Adjusted Logistic Regression Models Predicting Menthol or Capsule Cigarette Smoking From Survey Wave, Country, and Demographic Covariates

		Usual brand/variety of cigarettes is menthol or capsule (yes vs. other)			Smoked any menthol or capsule cigarettes in the past 30 days (yes vs. other)		
	**Sample n**	**%**	**AOR (95% CI)**	**p**	**%**	**AOR (95% CI)**	**p**
**SURVEY WAVE**							
Aug 2020	1531	11.9	REF		47.7	REF	
Feb 2020	2180	14.6	**1.42 (1.11-1.81)**	**.006**	55.5	1.41 (1.20-1.65)	**<.001**
Aug 2019	1693	13.9	1.27 (0.98-1.64)	.065	52.5	1.26 (1.06-1.49)	**.008**
Aug 2018	1663	15.4	1.23 (0.95-1.60)	.113	48.9	0.98 (0.83-1.16)	.831
**COUNTRY**							
Canada	2113	2.4	REF		36.4	REF	
England	2843	8.9	**4.07 (2.83-5.86)**	**<.001**	50.9	**2.06 (1.79-2.36)**	**<.001**
US	2111	34.6	**22.71 (15.92-32.39)**	**<.001**	66.6	**3.99 (3.40-4.69)**	**<.001**
**AGE GROUP**							
16-17	2786	14.1	REF		49.8	REF	
18-19	4281	14.0	1.06 (0.89-1.27)	.493	52.4	1.11 (0.98-1.25)	.087
**SEX**							
Male	2,938	13.7	REF		51.7	REF	
Female	4,129	14.4	**1.23 (1.03-1.46)**	**.019**	50.5	0.99 (0.88-1.10)	.811
**RACE/ETHNICITY**							
White only	5,019	14.1	REF		49.7	REF	
Any black	607	24.3	**1.92 (1.51-2.44)**	**<.001**	63.0	**1.79 (1.43-2.24)**	**<.001**
Any other or mixed	1,284	9.9	0.89 (0.70-1.14)	.358	53.4	**1.51 (1.29-1.78)**	**<.001**
Don't know/refused	157	8.2	1.93 (0.89-4.20)	.097	52.8	**2.14 (1.48-3.09)**	**<.001**

All data except sample n are weighted.

© 2022 East KA et al. *JAMA Network Open.*

eFigure 1. Percentage of Past 30-Day Smokers Who Reported That Had Smoked Any Menthol or Capsule Cigarettes in the Past 30 Days by Demographic Characteristics <u>in England Only</u> at Each Survey Wave (N=2,843)

a) Age group

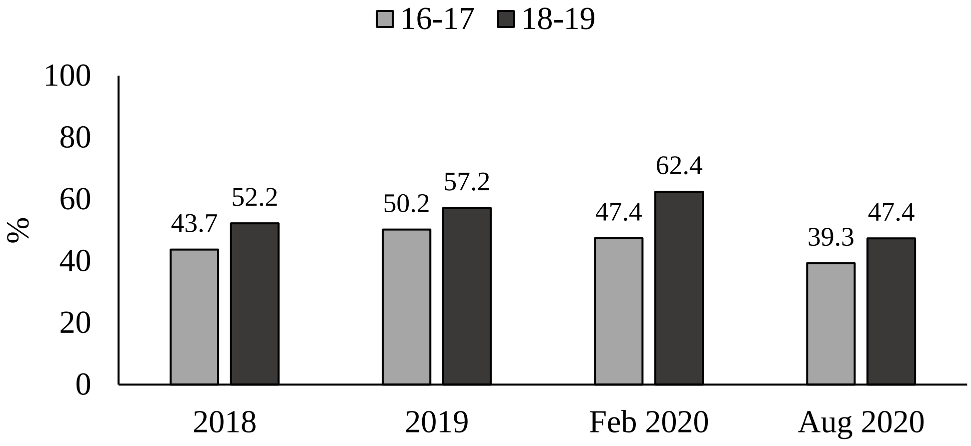

b) Sex

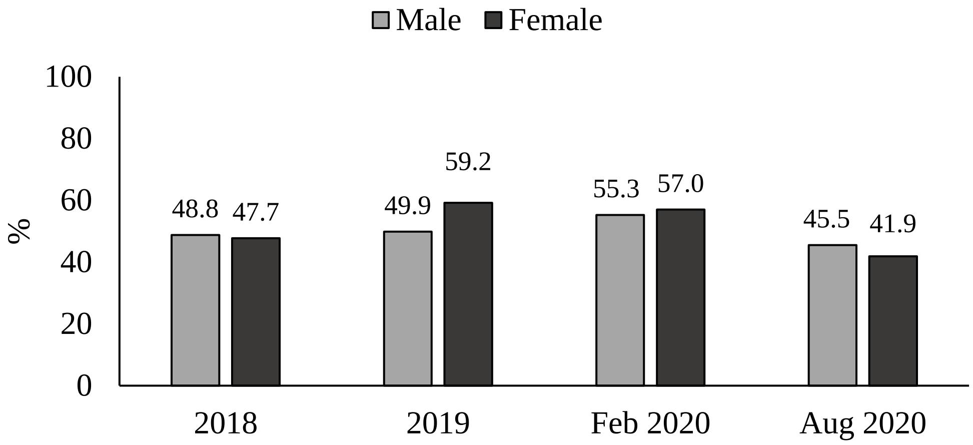

c) Race/ethnicity

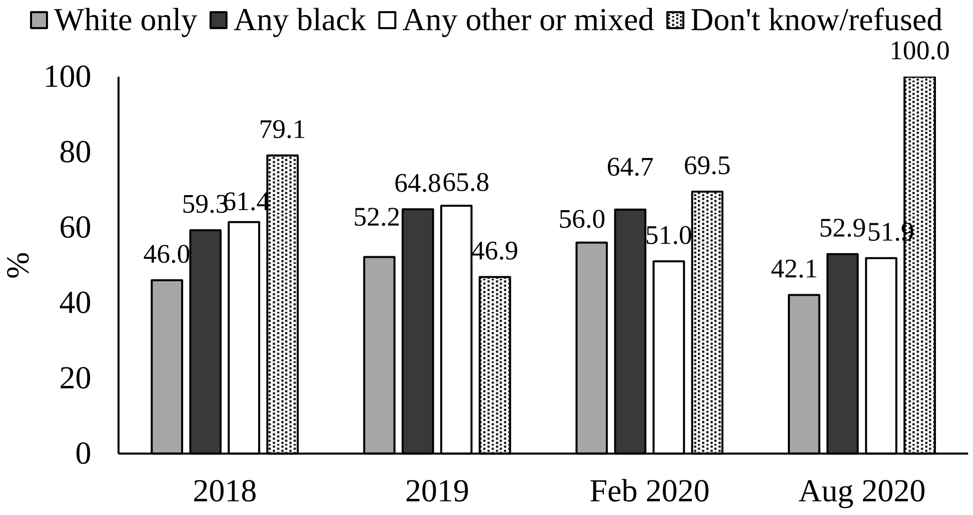

© 2022 East KA et al. *JAMA Network Open.*

eFigure 2. Percentage of Past 30-Day Smokers Who Reported That Had Smoked Any Menthol or Capsule Cigarettes in the Past 30 Days by Consumption/Dependence Indicators <u>in England Only</u> at Each Survey Wave (N=2,843)

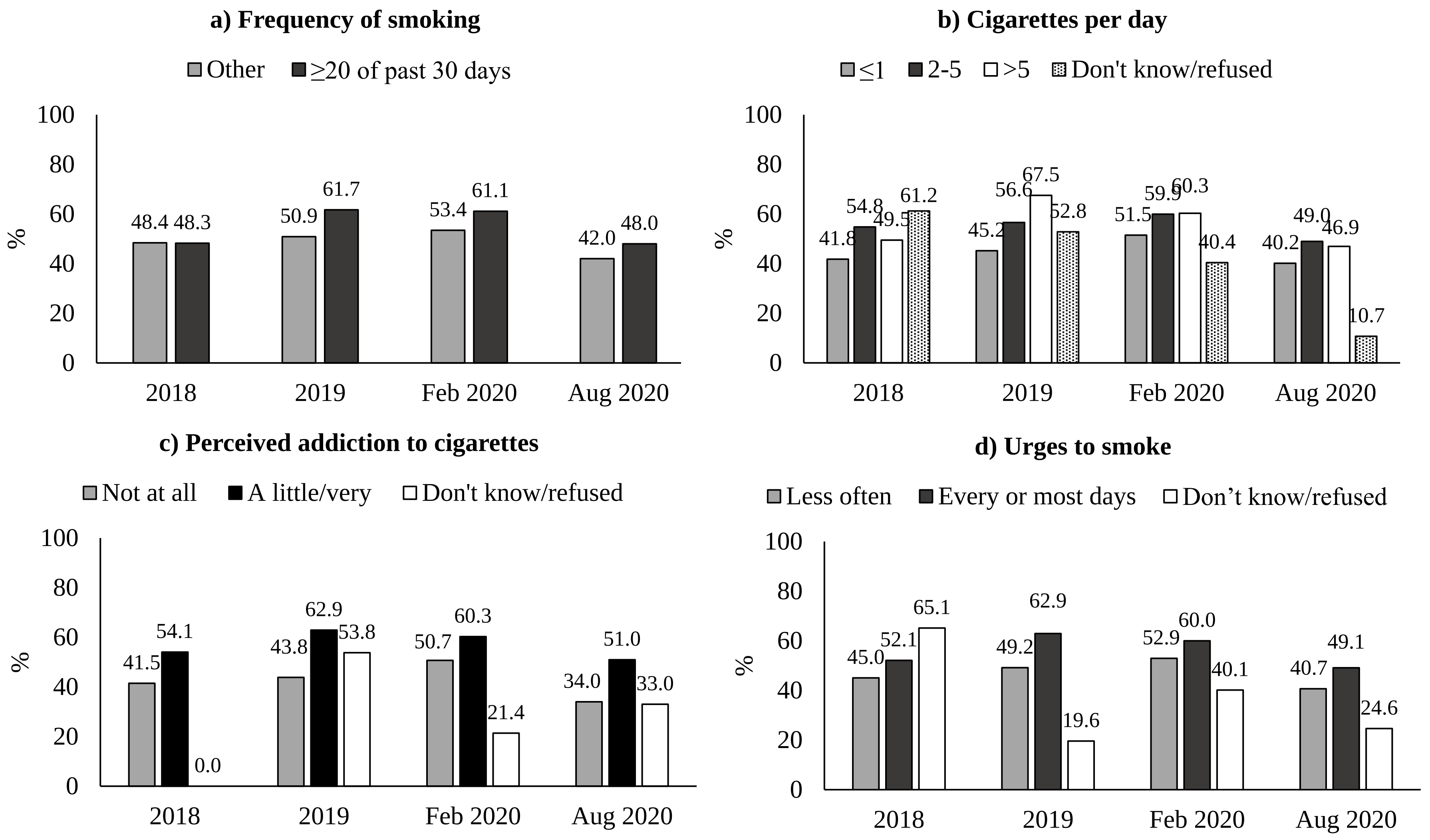

© 2022 East KA et al. *JAMA Network Open.*

eTable 7. Number and Proportion of Past 30-Day Smokers Who Reported a Usual Brand/Variety of Cigarettes That Was Menthol Only (Not Capsule) by Demographics, Frequent Smoking, Cigarettes Smoked per Day, Perceived Addiction, and Urges to Smoke, Split by Country and Survey Wave. Data are weighted % (unweighted n).

	CANADA				ENGLAND				US			
	Aug 2018 (N=584)	Aug 2019 (N=557)	Feb 2020 (N=614)	Aug 2020 (N=358)	Aug 2018 (N=634)	Aug 2019 (N=588)	Feb 2020 (N=936)	Aug 2020 (N=685)	Aug 2018 (N=445)	Aug 2019 (N=548)	Feb 2020 (N=630)	Aug 2020 (N=488)
**AGE GROUP**												
16-17	2.3 (5)	1.1 (3)	1.6 (3)	0.4 (1)	4.0 (7)	3.6 (6)	3.9 (14)	0.5 (1)	31.8 (68)	30.5 (67)	33.6 (111)	32.4 (88)
18-19	2.5 (7)	1.5 (5)	1.0 (3)	1.2 (2)	4.3 (18)	6.1 (24)	4.1 (27)	0.2 (2)	29.1 (68)	31.1 (119)	30.6 (108)	35.8 (77)
**SEX**												
Male	2.7 (7)	0.8 (2)	1.5 (3)	0.7 (1)	3.7 (10)	4.0 (8)	3.2 (13)	0.5 (2)	32.3 (73)	28.2 (64)	30.5 (84)	35.7 (78)
Female	2.1 (5)	2.0 (6)	1.1 (3)	1.0 (2)	4.8 (15)	6.2 (22)	5.0 (28)	0.1 (1)	27.4 (63)	34.8 (122)	34.6 (135)	30.7 (87)
**RACE/ETHNICITY**												
White only	2.0 (4)	1.7 (6)	1.5 (4)	0.6 (1)	4.2 (23)	5.0 (26)	4 (35)	0.2 (2)	27.0 (81)	28.5 (110)	26.6 (110)	33.1 (99)
Any black	0.0 (0)	1.8 (1)	2.5 (1)	0.0 (0)	6.3 (1)	11.4 (2)	2.2 (1)	0.0 (0)	61.8 (31)	48.6 (43)	68.6 (55)	50.9 (34)
Any other	2.3 (4)	0.4 (1)	0.5 (1)	1.5 (2)	0.0 (0)	4.2 (2)	5.7 (4)	2.2 (1)	25.7 (23)	34.7 (32)	38.4 (53)	29 (31)
Don't know/refused	5.3 (4)	0.0 (0)	0.0 (0)	0.0 (0)	25.7 (1)	0.0 (0)	7.9 (1)	0.0 (0)	46.1 (1)	26.3 (1)	30.9 (1)	60.9 (1)
**FREQUENT SMOKING**												
Other	3.1 (9)	1.7 (7)	1.9 (6)	1.1 (3)	5.4 (21)	5.5 (22)	4.5 (31)	0.5 (3)	26.2 (81)	30.1 (121)	31.0 (136)	32.8 (94)
≥20 of past 30 days	1.5 (3)	0.5 (1)	0.0 (0)	0.0 (0)	1.8 (4)	4.3 (8)	3.1 (10)	0.0 (0)	38.7 (55)	32.4 (65)	34.4 (83)	36.2 (71)
**CIGARETTES PER DAY**												
≤1	2.8 (6)	1.7 (5)	1.3 (3)	1.1 (2)	6.4 (14)	5.8 (13)	5.1 (23)	0.4 (1)	26.9 (52)	30.3 (74)	30.7 (91)	25.3 (59)
2 to 5	1.9 (3)	1.2 (2)	2.0 (3)	0.9 (1)	3.3 (8)	5.3 (12)	3.4 (12)	0.5 (2)	31.6 (46)	34.1 (67)	34.1 (80)	39.6 (59)
>5	2.6 (3)	0.6 (1)	0.0 (0)	0.0 (0)	0.7 (2)	3.6 (5)	3.2 (6)	0.0 (0)	34.9 (34)	27.6 (43)	32.3 (47)	41.0 (47)
Don't know/refused	0.0 (0)	0.0 (0)	0.0 (0)	0.0 (0)	17.1 (1)	0.0 (0)	0.0 (0)	0.0 (0)	50.3 (4)	21.7 (2)	18.1 (1)	0.0 (0)
**PERCEIVED ADDICTION**												
Not at all	2.9 (5)	1.7 (4)	0.0 (0)	0.0 (0)	4.9 (14)	6.0 (13)	5.2 (19)	0.6 (2)	32.6 (51)	25.6 (50)	26.3 (61)	32.5 (50)
A little/very	2.2 (7)	1.1 (4)	2.0 (6)	1.4 (3)	3.5 (11)	4.4 (17)	3.4 (22)	0.2 (1)	29.2 (82)	33.7 (134)	34.4 (153)	34.2 (111)
Don't know/refused	0.0 (0)	0.0 (0)	0.0 (0)	0.0 (0)	0.0 (0)	0.0 (0)	0.0 (0)	0.0 (0)	32.3 (3)	7.4 (2)	60.6 (5)	78.2 (4)
**URGES TO SMOKE**												
Less often	3.2 (7)	1.6 (6)	1.4 (4)	0.0 (0)	4.7 (16)	4.7 (17)	3.7 (20)	0.4 (1)	25.6 (51)	27.0 (70)	29.3 (90)	31.3 (65)
Every or most days	1.7 (5)	1.1 (2)	1.1 (2)	1.9 (3)	3.2 (8)	5.7 (13)	4.5 (21)	0.3 (2)	34.5 (82)	33.2 (115)	35.3 (128)	36.6 (100)
Don’t know/refused	0.0 (0)	0.0 (0)	0.0 (0)	0.0 (0)	16.8 (1)	0.0 (0)	0.0 (0)	0.0 (0)	40.6 (3)	49.9 (1)	6.4 (1)	0.0 (0)

© 2022 East KA et al. *JAMA Network Open.*

eTable 8. Number and Proportion of Past 30-Day Smokers Who Report Smoking Any Menthol Only (Not Capsule) Cigarettes in the Past 30 Days by Demographics, Frequent Smoking, Cigarettes Smoked per Day, Perceived Addiction, and Urges to Smoke, Split by Country and Survey Wave. Data are weighted % (unweighted n).

	CANADA				ENGLAND				US			
	Aug 2018 (N=584)	Aug 2019 (N=557)	Feb 2020 (N=614)	Aug 2020 (N=358)	Aug 2018 (N=634)	Aug 2019 (N=588)	Feb 2020 (N=936)	Aug 2020 (N=685)	Aug 2018 (N=445)	Aug 2019 (N=548)	Feb 2020 (N=630)	Aug 2020 (N=488)
**AGE GROUP**												
16-17	27.7 (63)	32.5 (57)	33.2 (81)	30.1 (52)	37.2 (66)	46.9 (93)	38.9 (140)	31.7 (64)	60.9 (131)	64.6 (139)	67.1 (209)	64.3 (168)
18-19	33.6 (108)	30.0 (102)	35.1 (124)	29.8 (50)	47.5 (219)	52.6 (212)	56.8 (329)	41.0 (201)	50.8 (120)	64.1 (224)	66.1 (203)	58.7 (139)
**SEX**												
Male	33.7 (104)	33.6 (70)	33.1 (76)	32.7 (50)	41.3 (108)	46.9 (100)	46.8 (169)	37.0 (104)	54.4 (118)	61.5 (127)	64.5 (168)	62.3 (149)
Female	26.1 (67)	27.4 (89)	35.7 (129)	26.7 (52)	44.9 (177)	54.0 (205)	52.0 (300)	37.1 (161)	57.6 (133)	68.5 (236)	69.9 (244)	60.0 (158)
**RACE/ETHNICITY**												
White only	20.4 (56)	29.8 (87)	28.5 (110)	23.0 (51)	40.6 (228)	47.8 (244)	48.9 (393)	35.9 (214)	54.8 (163)	63.1 (236)	66.1 (276)	60.3 (198)
Any black	40.9 (16)	33.2 (17)	46.5 (20)	31.8 (6)	57.7 (18)	64.8 (18)	55.0 (31)	36.5 (20)	63.9 (39)	68.9 (56)	73.3 (55)	70.2 (44)
Any other	32.4 (56)	34.8 (53)	44.7 (70)	45.2 (44)	52.7 (35)	63.1 (40)	47.2 (40)	45.7 (28)	54.4 (48)	69.6 (69)	65.8 (80)	65.0 (64)
Don't know/refused	54.5 (43)	8.3 (2)	47.8 (5)	16.5 (1)	79.1 (4)	41.7 (3)	69.5 (5)	100.0 (3)	53.9 (1)	59.0 (2)	30.9 (1)	60.9 (1)
**FREQUENT SMOKING**												
Other	24.4 (82)	32.0 (112)	34.1 (141)	31.5 (77)	43.7 (192)	47.4 (194)	46.1 (283)	34.9 (165)	54.1 (166)	64.9 (239)	64.3 (265)	59.0 (186)
≥20 of past 30 days	39.5 (89)	28.5 (47)	34.7 (64)	25.5 (25)	41.3 (93)	56.3 (111)	55.1 (186)	41.5 (100)	58.7 (85)	63.1 (124)	71.6 (147)	65.4 (121)
**CIGARETTES PER DAY**												
≤1	26.0 (62)	32.3 (77)	33.1 (95)	30.7 (53)	38.3 (108)	41.1 (99)	42.7 (171)	34.1 (102)	52.0 (104)	66.2 (151)	58.9 (167)	52.2 (114)
2 to 5	35.5 (64)	33.5 (49)	41.3 (84)	31.9 (34)	47.3 (123)	52.5 (113)	54.7 (173)	41.5 (96)	56.3 (81)	64.7 (123)	73.9 (147)	71.7 (112)
>5	33.8 (45)	25.6 (32)	26.4 (26)	25.5 (15)	43.5 (52)	63.5 (89)	54.1 (119)	38.7 (65)	61.9 (61)	59.6 (86)	72.7 (97)	64.1 (80)
Don't know/refused	0.0 (0)	16.3 (1)	0.0 (0)	0.0 (0)	61.2 (2)	52.8 (4)	40.4 (6)	10.7 (2)	69.4 (5)	69.5 (3)	18.1 (1)	12.8 (1)
**PERCEIVED ADDICTION**												
Not at all	20.4 (40)	31.4 (62)	26.8 (53)	24.4 (32)	38.9 (132)	41.8 (111)	43.5 (157)	30.2 (92)	49.0 (83)	62.3 (102)	55.9 (118)	51.1 (82)
A little/very	37.3 (130)	31.4 (97)	38.1 (147)	32.9 (68)	46.1 (153)	57.1 (189)	53.4 (309)	41.8 (170)	59.3 (164)	66.3 (259)	71.5 (289)	66.2 (221)
Don't know/refused	7.8 (1)	0.0 (0)	39.3 (5)	62.2 (2)	0.0 (0)	53.8 (5)	21.4 (3)	33.0 (3)	52.3 (4)	6.8 (2)	70.0 (5)	78.2 (4)
**URGES TO SMOKE**												
Less often	23.5 (65)	28.9 (82)	30.8 (96)	31.3 (61)	41.5 (168)	45.6 (159)	46.4 (225)	34.8 (130)	44.4 (92)	65.9 (139)	58.4 (164)	52.9 (121)
Every or most days	38.6 (106)	34.0 (75)	39.3 (108)	28.7 (41)	44.1 (114)	58.1 (145)	53.2 (242)	40.9 (132)	65.4 (155)	62.8 (222)	74.9 (244)	69.2 (186)
Don’t know/refused	0.0 (0)	26.6 (2)	10.6 (1)	0.0 (0)	65.1 (3)	19.6 (1)	8.0 (2)	19.0 (3)	72.0 (4)	100.0 (2)	33.7 (4)	0.0 (0)

© 2022 East KA et al. *JAMA Network Open.*

eTable 9. Contrasts Within Countries for the Percentage of Past 30-Day Smokers Who Smoked Menthol Only (Not Capsule) Cigarettes by Survey Wave (N=7,067)

	Usual brand/variety of cigarettes is menthol (yes vs. other)			Smoked any menthol cigarettes in the past 30 days (yes vs. other)		
	**%**	**AOR (95% CI)**	**p**	**%**	**AOR (95% CI)**	**p**
**ENGLAND**						
Aug 2020	0.3	REF		37.1	REF	
Feb 2020	4.0	**1.04 (1.02-1.06)**	**<.001**	49.2	**1.06 (1.00-1.13)**	**.048**
2019	5.1	**1.05 (1.03-1.07)**	**<.001**	50.3	**1.14 (1.07-1.21)**	**<.001**
2018	4.1	**1.04 (1.02-1.05)**	**<.001**	42.8	**1.13 (1.07-1.19)**	**<.001**
**CANADA**						
Aug 2020	0.8	REF		29.9	REF	
Feb 2020	1.3	1.01 (1.00-1.03)	.120	34.3	0.98 (0.92-1.05)	.586
Aug 2019	1.3	1.00 (0.99-1.02)	.496	30.9	1.00 (0.94-1.07)	.910
Aug 2018	2.4	1.00 (0.99-1.02)	.531	30.7	1.04 (0.98-1.11)	.197
**US**						
Aug 2020	34.1	REF		61.5	REF	
Feb 2020	32.1	0.96 (0.89-1.03)	.224	66.6	0.93 (0.86-1.01)	.093
Aug 2019	30.8	0.96 (0.90-1.04)	.334	64.3	1.02 (0.95-1.11)	.572
Aug 2018	30.4	0.97 (0.90-1.05)	.493	55.6	1.04 (0.97-1.13)	.267

Data are weighted. Contrasts (AOR, 95% CI, p) are derived from interactions from logistic regression models adjusted for age group, sex, and race/ethnicity.

© 2022 East KA et al. *JAMA Network Open.*

eTable 10. Contrasts Within Countries for the Percentage of Past 30-Day Smokers Who Smoked Menthol Only (Not Capsule) Cigarettes by Demographics (N=7,067)

	Usual brand/variety of cigarettes is menthol (yes vs. other)			Smoked any menthol cigarettes in the past 30 days (yes vs. other)		
	%	AOR (95% CI)	p	%	AOR (95% CI)	p
**CANADA**						
AGE						
16-17	1.4	REF		30.7	REF	
18-19	1.5	1.00 (0.99-1.01)	.879	32.1	1.00 (0.96-1.05)	.911
SEX						
Male	1.4	REF		33.3	REF	
Female	1.5	1.00 (0.99-1.01)	.752	29.1	0.96 (0.92-1.00)	.052
RACE/ETHNICITY						
White only	1.4	REF		25.8	REF	
Any black	1.1	1.00 (0.98-1.01)	.706	38.1	**1.13 (1.03-1.23)**	**.007**
Any other or mixed	1.2	1.00 (0.99-1.01)	.704	39.0	**1.14 (1.08-1.20)**	**<.001**
Don't know/refused	3.7	1.02 (0.99-1.06) [2]	.219	45.3	**1.25 (1.13-1.38)** [2]	**<.001**
**ENGLAND**						
AGE						
16-17	3.0	REF		38.2	REF	
18-19	3.6	1.00 (0.99-1.02)	.657	49.9	**1.11 (1.07-1.16)**	**<.001**
SEX						
Male	2.8	REF		42.9	REF	
Female	4.0	1.01 (1.00 -1.03)	.110	47.3	1.04 (1.00-1.08)	.052
RACE/ETHNICITY						
White only	3.3	REF		43.5	REF	
Any black	3.7	1.00 (0.97-1.04)	.814	51.0	1.07 (0.98-1.17)	.127
Any other or mixed	3.2	1.00 (0.97-1.02)	.938	52.1	**1.09 (1.01-1.17)**	**.023**
Don't know/refused	10.2	1.07 (0.92-1.25)	.388	67.6	**1.27 (1.05-1.54)**	**.016**
**US**						
AGE						
16-17	32.1	REF		64.0	REF	
18-19	31.5	0.99 (0.94-1.05)	.791	59.2	0.95 (0.90-1.00)	.071
SEX						
Male	32.0	REF		60.3	REF	
Female	31.5	0.99 (0.95-1.04)	.818	63.6	1.03 (0.98-1.08)	.243
RACE/ETHNICITY						
White only	28.8	REF		60.6	REF	
Any black	58.5	**1.35 (1.25-1.45)**	**<.001**	68.5	**1.08 (1.00-1.17)**	**.043**
Any other or mixed	31.3	1.02 (0.96-1.09)	.450	62.2	1.01 (0.95-1.09)	.682
Don't know/refused	41.0	1.13 (0.74-1.72) [2]	.570	48.2	0.89 (0.57-1.41) [2]	.627

Data are weighted. Contrasts (AOR, 95% CI, p) are derived from interactions from logistic regression models adjusting for country, survey wave, age, sex, and race/ethnicity. Data are aggregated across survey waves.
[1] Estimate unreportable due to n=0. [2] Treat estimate with caution (denominator n<30).

© 2022 East KA et al. *JAMA Network Open.*

eTable 11. Contrasts Within Countries for the Percentage of Past 30-Day Smokers Who Smoked Menthol Only (Not Capsule) Cigarettes by Consumption/Dependence Indicators (N=7,067)

	Usual brand/variety of cigarettes is menthol (yes vs. other)			Smoked any menthol cigarettes in the past 30 days (yes vs. other)		
	**%**	**AOR (95% CI)**	**p**	**%**	**AOR (95% CI)**	**p**
**CANADA**						
FREQUENT SMOKING						
Other	1.9	REF		30.8	REF	
≥20 of past 30 days	0.6	**0.99 (0.98-0.996)**	**.005**	33.0	1.02 (0.98-1.07)	.378
CIGARETTES PER DAY						
≤1	1.7	REF		30.7	REF	
2-5	1.5	1.00 (0.99-1.01)	.724	35.8	1.05 (1.00-1.10)	.063
>5	0.9	0.99 (0.98-1.00)	.164	28.2	0.97 (0.92-1.02)	.266
Don't know/refused[1]	0.0	-[1,2]	-	3.9	**0.77 (0.72-0.82)** [2]	**<.001**
PERCEIVED ADDICTION						
Not at all	1.2	REF		25.9	REF	
A little/very	1.7	1.00 (0.99-1.01)	.466	35.0	**1.09 (1.04-1.14)**	**<.001**
Don't know/refused[1]	0.0	-[1,2]		24.9	0.97 (0.82-1.15) [2]	.746
URGES TO SMOKE						
Less often	1.5	REF		28.8	REF	
Every or most days	1.5	1.00 (0.99-1.01)	.812	35.4	**1.07 (1.02-1.11)**	**.003**
Don't know/refused[1]	0.0	-[1,2]		10.7	**0.84 (0.75-0.93)** [2]	**.001**
**ENGLAND**						
FREQUENT SMOKING						
Other	3.9	REF		43.0	REF	
≥20 of past 30 days	2.3	**0.98 (0.97-1.00)**	**.037**	48.6	**1.07 (1.02-1.12)**	**.003**
CIGARETTES PER DAY						
≤1	4.3	REF		39.1	REF	
2-5	3.1	0.99 (0.97-1.01)	.169	49.3	**1.11 (1.06-1.16)**	**<.001**
>5	1.9	**0.98 (0.96-0.99)**	**.010**	49.8	**1.12 (1.06-1.18)**	**<.001**
Don't know/refused	2.0	0.98 (0.94-1.02)	.288	35.9	0.97 (0.82-1.14)	.676
PERCEIVED ADDICTION						
Not at all	4.2	REF		38.8	REF	
A little/very	2.8	0.99 (0.97-1)	.074	49.5	**1.12 (1.07-1.17)**	**<.001**
Don't know/refused	0.0	-[1,2]		33.7	0.96 (0.79-1.15) [2]	.648
URGES TO SMOKE						
Less often	3.3	REF		42.2	REF	
Every or most days	3.4	1.00 (0.99-1.02)	.941	48.9	**1.08 (1.03-1.12)**	**<.001**
Don't know/refused	2.7	0.99 (0.94-1.05) [2]	.809	23.3	0.84 (0.71-0.99)	.036
**US**						
FREQUENT SMOKING						
Other	29.7	REF		60.1	REF	
≥20 of past 30 days	35.8	**1.07 (1.02-1.13)**	**.010**	64.3	1.05 (1.00-1.12)	.069
CIGARETTES PER DAY						
≤1	28.2	REF		56.8	REF	
2-5	34.7	**1.07 (1.01-1.13)**	**.023**	66.3	**1.10 (1.04-1.17)**	**.002**
>5	34.7	**1.08 (1.01-1.15)**	**.034**	64.4	**1.09 (1.02-1.17)**	**.017**
Don't know/refused	30.6	1.00 (0.81-1.23) [2]	.965	52.0	0.96 (0.72-1.28) [2]	.768
PERCEIVED ADDICTION						
Not at all	29.7	REF		53.8	REF	
A little/very	32.6	1.03 (0.97-1.09)	.367	65.5	**1.13 (1.06-1.20)**	**<.001**
Don't know/refused	42.1	1.12 (0.91-1.39) [2]	.279	52.1	0.99 (0.78-1.25) [2]	.930
URGES TO SMOKE						
Less often	28.2	REF		54.0	REF	
Every or most days	34.9	**1.06 (1.01-1.12)**	**.024**	67.9	**1.15 (1.09-1.22)**	**<.001**
Don't know/refused	28.7	0.98 (0.78-1.22) [2]	.824	60.1	1.05 (0.78-1.40) [2]	.759

Data are weighted. Contrasts (AOR, 95% CI, p) are derived from interactions from logistic regression models adjusting for country, survey wave, age, sex, and race/ethnicity. Data are aggregated across survey waves.
[1] Estimate unreportable due to n=0. [2] Treat estimate with caution (denominator n<30).

© 2022 East KA et al. *JAMA Network Open.*

eTable 12. Adjusted Logistic Regression Models Predicting Menthol Only (Not Capsule) Cigarette Smoking From Survey Wave, Country, and Demographic Covariates

	Sample n	Usual brand/variety of cigarettes is menthol (yes vs. other)			Smoked any menthol cigarettes in the past 30 days (yes vs. other)		
		%	AOR (95% CI)	p	%	AOR (95% CI)	p
**SURVEY WAVE**							
Aug 2020	1531	9.5	REF		41.7	REF	
Feb 2020	2180	9.8	1.18 (0.89-1.58)	.251	49.6	**1.41 (1.20-1.66)**	**<.001**
Aug 2019	1693	10.6	1.20 (0.89-1.62)	.236	47.9	**1.32 (1.11-1.57)**	**.001**
Aug 2018	1663	11.9	1.12 (0.83-1.51)	.462	43.8	1.01 (0.85-1.20)	.887
**COUNTRY**							
Canada	2113	1.5	REF		31.5	REF	
England	2843	3.4	**2.63 (1.65-4.19)**	**<.001**	44.9	**2.00 (1.74-2.30)**	**<.001**
US	2111	31.8	**35.16 (23.01-53.73)**	**<.001**	61.5	**4.03 (3.43-4.73)**	**<.001**
**AGE GROUP**							
16-17	2786	11.0	REF		43.7	REF	
18-19	4281	10.0	1.00 (0.81-1.23)	.993	47.5	**1.17 (1.04-1.32)**	**.009**
**SEX**							
Male	2,938	11.0	REF		45.5	REF	
Female	4,129	9.8	1.07 (0.87-1.3)	.526	46.2	1.07 (0.96-1.20)	.246
**RACE/ETHNICITY**							
White only	5,019	9.9	REF		44.6	REF	
Any black	607	22.7	**2.69 (2.05-3.54)**	**<.001**	53.7	1.46 (1.18-1.81)	**.001**
Any other or mixed	1,284	8.2	1.08 (0.82-1.41)	.598	47.8	1.46 (1.25-1.72)	**<.001**
Don't know/refused	157	6.6	**2.97 (1.15-7.62)**	**.024**	50.0	2.35 (1.62-3.40)	**<.001**

All data except sample n are weighted.

© 2022 East KA et al. *JAMA Network Open.*

eFigure 3. Percentage of Past 30-Day Smokers Who Reported a Usual Brand/Variety of Cigarettes That Was Menthol Only (Not Capsule) by Demographic Characteristics in England at Each Wave (N=2,843)

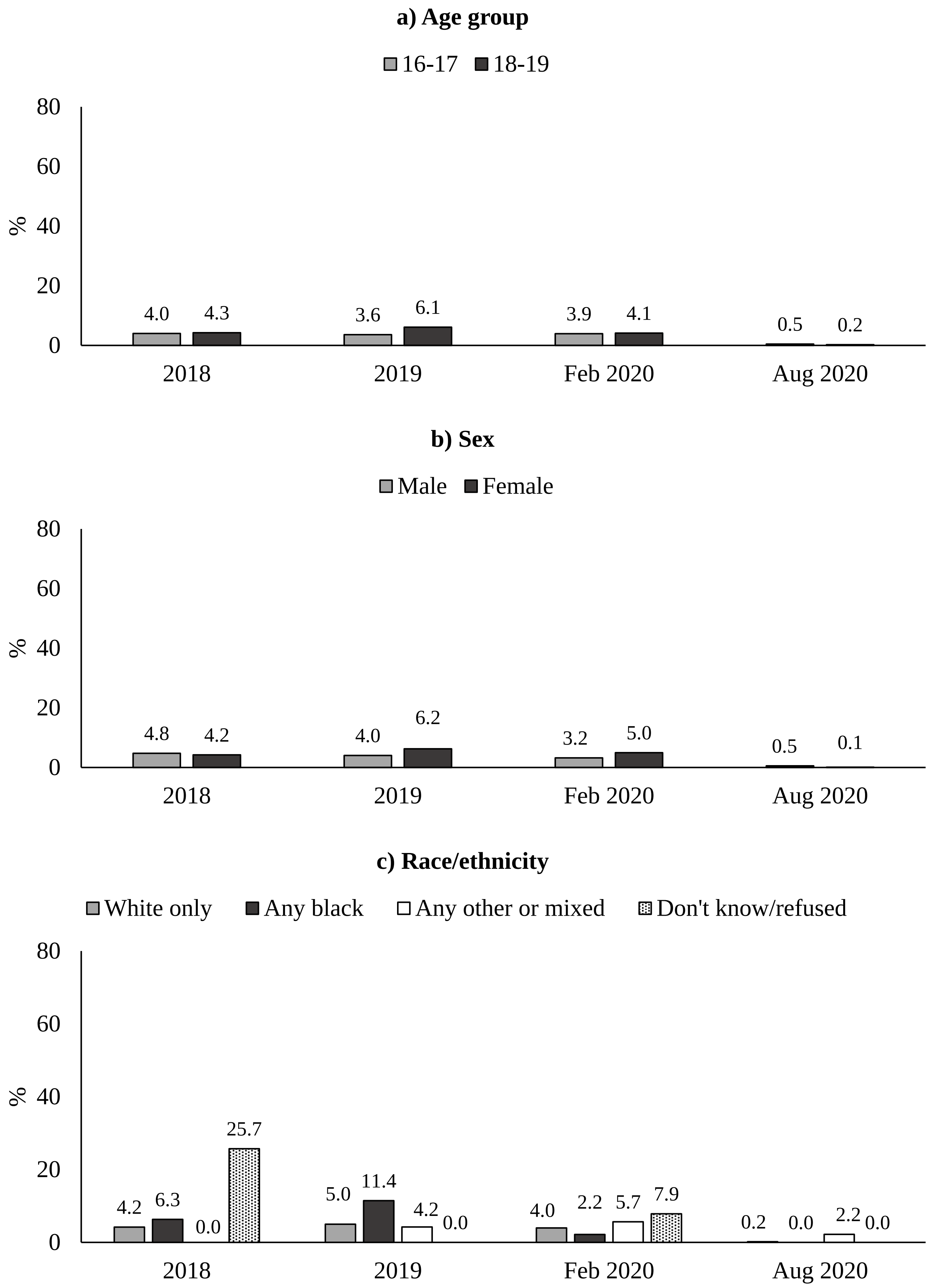

© 2022 East KA et al. *JAMA Network Open.*

eFigure 4. Percentage of Past 30-Day Smokers Who Reported That They Had Smoked Any Menthol Only (Not Capsule) Cigarettes by Demographic Characteristics in England at Each Wave (N=2,843)

a) Age group

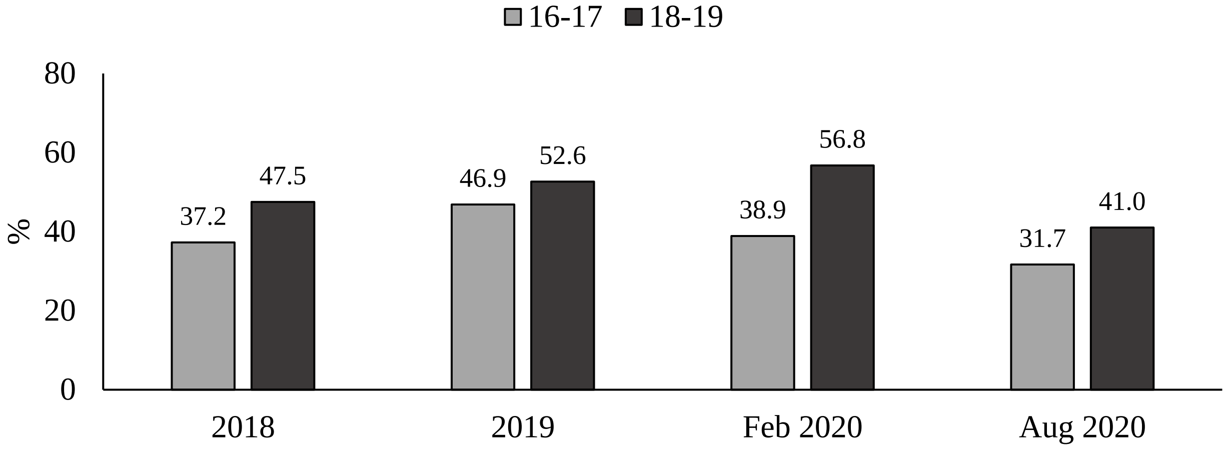

b) Sex

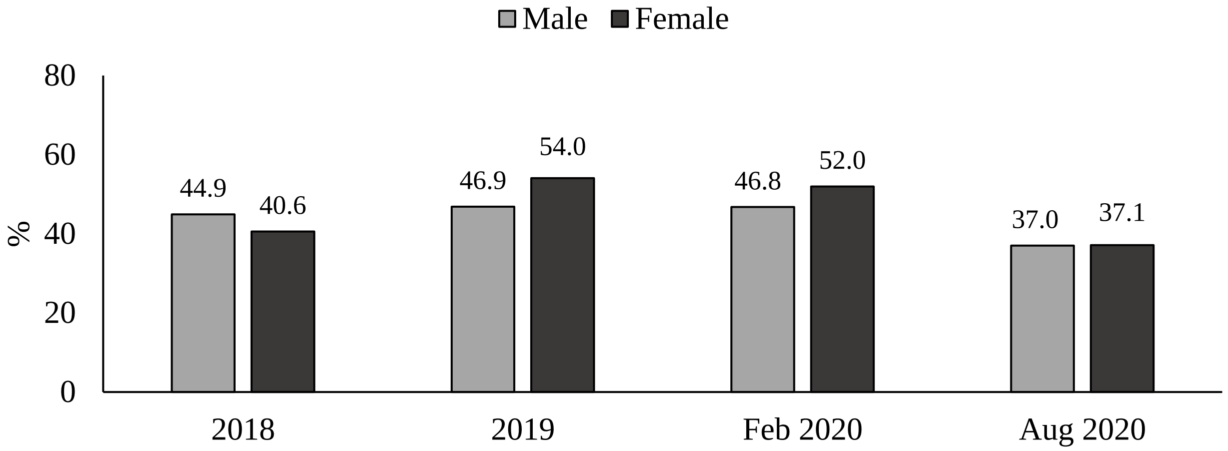

c) Race/ethnicity

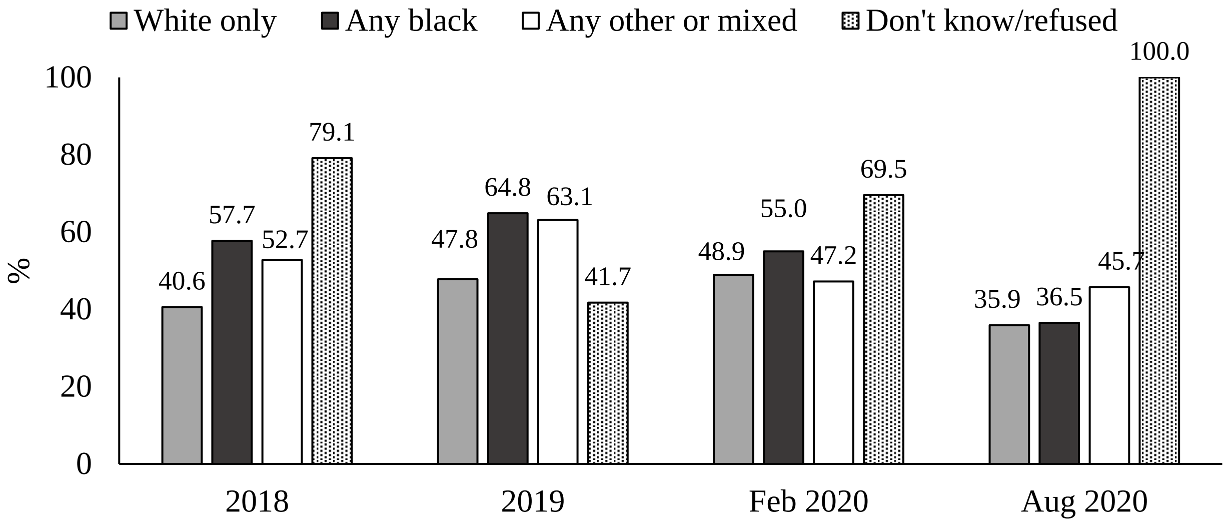

© 2022 East KA et al. *JAMA Network Open.*

eFigure 5. Percentage of Past 30-Day Smokers Who Reported a Usual Brand/Variety of Cigarettes That Was Menthol Only (Not Capsule) by Consumption/Dependence Indicators in England at Each Wave (N=2,843)

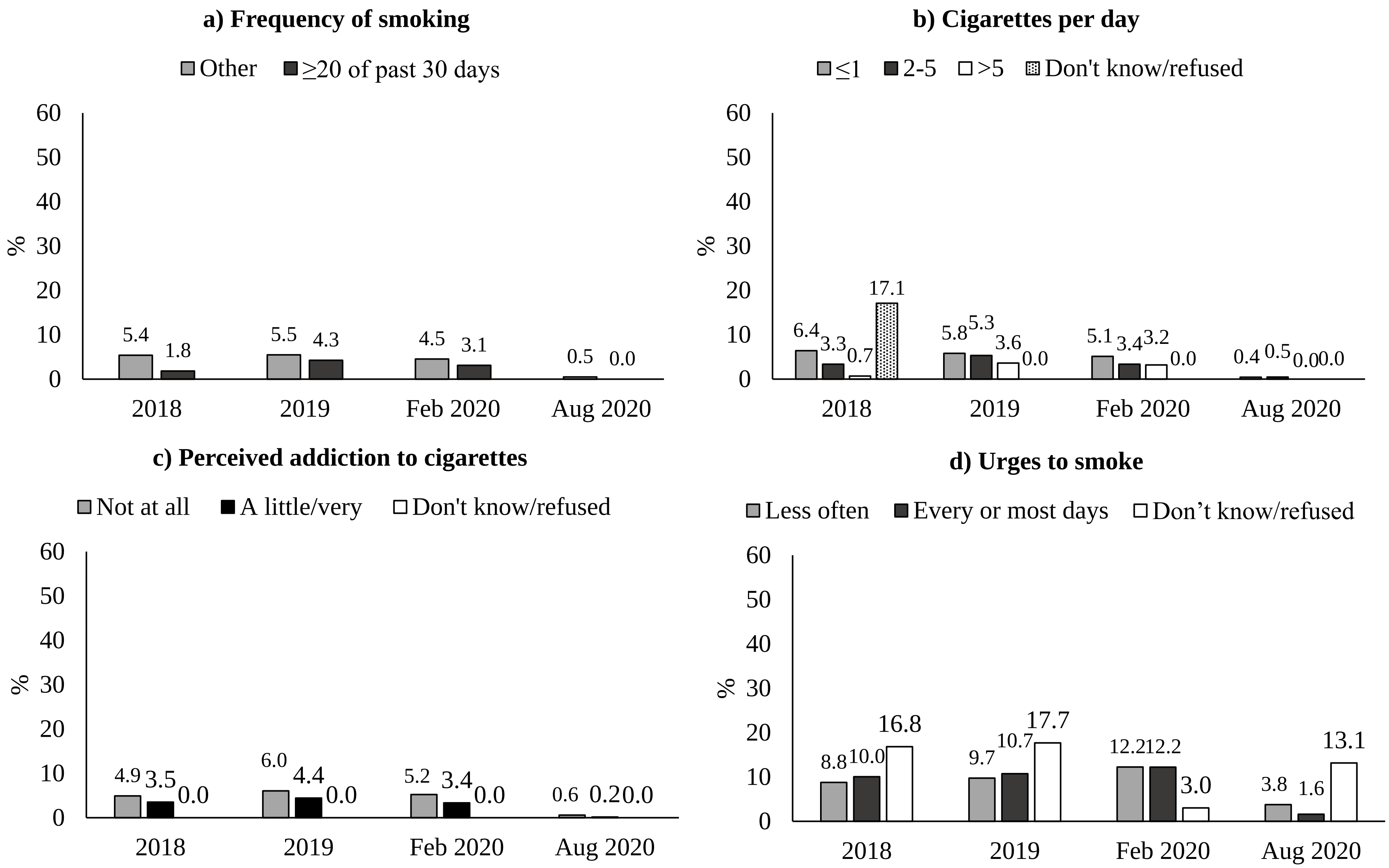

© 2022 East KA et al. *JAMA Network Open.*

eFigure 6. Percentage of Past 30-Day Smokers Who Reported That They Had Smoked Any Menthol Only (Not Capsule) Cigarettes in the Past 30 Days by Consumption/Dependence Indicators in England at Each Wave (N=2,843)

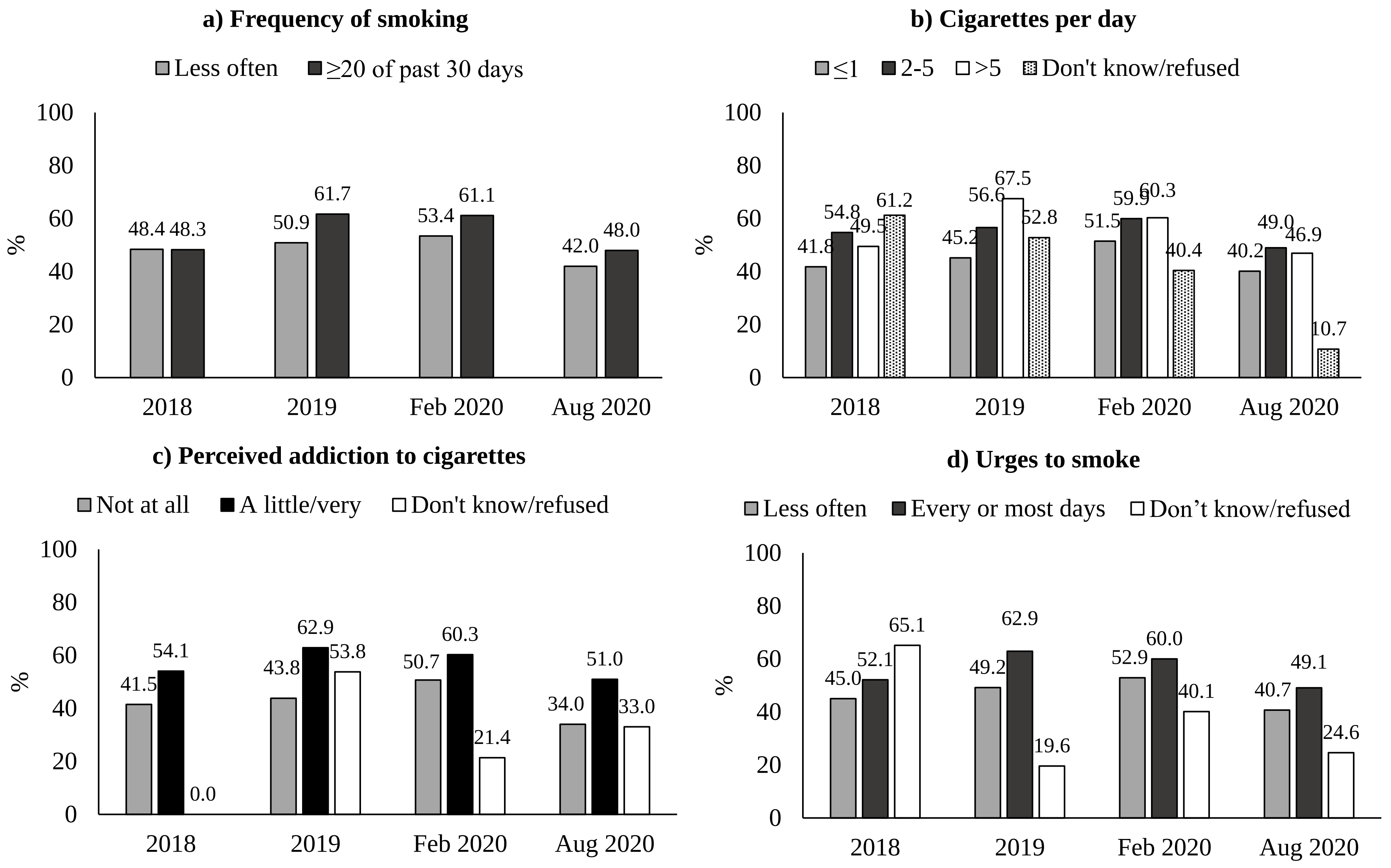

© 2022 East KA et al. *JAMA Network Open.*

eTable 13. Number and Proportion of Past 30-Day Smokers Who Reported a Usual Brand/Variety of Cigarettes That Was Capsule (Not Menthol Only) by Demographics, Frequent Smoking, Cigarettes Smoked per Day, Perceived Addiction, and Urges to Smoke, Split by Country and Survey Wave. Data are weighted % (unweighted n).

	CANADA				ENGLAND				US			
	Aug 2018 (N=584)	Aug 2019 (N=557)	Feb 2020 (N=614)	Aug 2020 (N=358)	Aug 2018 (N=634)	Aug 2019 (N=588)	Feb 2020 (N=936)	Aug 2020 (N=685)	Aug 2018 (N=445)	Aug 2019 (N=548)	Feb 2020 (N=630)	Aug 2020 (N=488)
**AGE GROUP**												
16-17	0.3 (1)	1.2 (2)	0.9 (2)	1.6 (2)	4.5 (8)	2.8 (6)	7.7 (27)	1.9 (4)	3.4 (8)	2.8 (7)	1.2 (3)	3.3 (8)
18-19	1.0 (3)	1.1 (4)	0.0 (0)	1.5 (2)	5.9 (30)	6.7 (30)	8.4 (49)	3.3 (19)	3.1 (5)	2.9 (15)	2.8 (11)	2.3 (7)
**SEX**												
Male	0.6 (2)	1.6 (4)	0.7 (2)	2.4 (3)	4.7 (12)	3.7 (8)	7.5 (26)	0.7 (2)	2.1 (3)	2.2 (6)	0.0 (0)	2.0 (4)
Female	0.8 (2)	0.6 (2)	0.0 (0)	0.4 (1)	5.9 (26)	6.7 (28)	8.8 (50)	5.2 (21)	5.1 (10)	3.8 (16)	5.3 (14)	4.4 (11)
**RACE/ETHNICITY**												
White only	0.8 (2)	1.5 (5)	0.6 (2)	2.1 (3)	5.8 (34)	5.1 (30)	8.6 (67)	3.0 (21)	3.7 (9)	2.8 (16)	2.5 (13)	3.1 (10)
Any black	0.0 (0)	0.0 (0)	0.0 (0)	0.0 (0)	3.1 (2)	3.4 (2)	4.1 (3)	0.0 (0)	3.5 (3)	2.7 (1)	0.0 (0)	1.3 (2)
Any other	1.1 (2)	0.8 (1)	0.0 (0)	0.7 (1)	1.8 (2)	4.7 (3)	6.3 (6)	0.7 (1)	0.8 (1)	3.2 (5)	0.6 (1)	1.7 (3)
Don't know/refused	0.0 (0)	0.0 (0)	0.0 (0)	0.0 (0)	0.0 (0)	16.8 (1)	0.0 (0)	22.5 (1)	0.0 (0)	0.0 (0)	0.0 (0)	0.0 (0)
**FREQUENT SMOKING**												
Other	0.6 (2)	1.2 (4)	0.2 (1)	1.3 (3)	5.7 (26)	4.6 (21)	8.1 (45)	3.0 (16)	4.0 (11)	2.8 (15)	2.5 (12)	2.5 (10)
≥20 of past 30 days	0.8 (2)	1.0 (2)	0.7 (1)	2.1 (1)	4.4 (12)	6.1 (15)	8.0 (31)	2.0 (7)	1.7 (2)	3.1 (7)	0.9 (2)	3.3 (5)
**CIGARETTES PER DAY**												
≤1	0.5 (1)	1.2 (3)	0.0 (0)	1.5 (2)	3.4 (12)	5.0 (13)	6.7 (28)	1.7 (5)	1.0 (3)	2.4 (9)	1.8 (5)	1.7 (6)
2 to 5	1.0 (2)	1.5 (2)	0.7 (1)	0.7 (1)	6.0 (17)	4.4 (12)	12.2 (32)	4.3 (14)	2.1 (5)	2.7 (7)	3.6 (9)	4.4 (6)
>5	0.6 (1)	0.6 (1)	1.0 (1)	3.0 (1)	7.9 (9)	6.6 (11)	5.5 (16)	2.7 (4)	7.7 (4)	4.2 (6)	0.0 (0)	2.5 (3)
Don't know/refused	0.0 (0)	0.0 (0)	0.0 (0)	0.0 (0)	0.0 (0)	0.0 (0)	0.0 (0)	0.0 (0)	32.5 (1)	0.0 (0)	0.0 (0)	0.0 (0)
**PERCEIVED ADDICTION**												
Not at all	0.5 (1)	0.0 (0)	0.0 (0)	0.0 (0)	3.2 (12)	5.9 (17)	7.5 (27)	4.4 (13)	2.4 (6)	3.6 (9)	3.5 (7)	2.1 (5)
A little/very	0.8 (3)	1.9 (6)	0.6 (2)	2.5 (4)	6.9 (26)	4.2 (18)	8.6 (49)	1.6 (10)	3.0 (6)	2.6 (13)	1.1 (6)	3.2 (10)
Don't know/refused	0.0 (0)	0.0 (0)	0.0 (0)	0.0 (0)	0.0 (0)	13.6 (1)	0.0 (0)	0.0 (0)	27.6 (1)	0.0 (0)	16.5 (1)	0.0 (0)
**URGES TO SMOKE**												
Less often	0.3 (1)	0.9 (2)	0.0 (0)	0.6 (1)	4.1 (20)	5.0 (19)	8.5 (38)	3.4 (16)	2.5 (7)	2.3 (10)	2.7 (8)	0.9 (3)
Every or most days	1.0 (3)	1.5 (4)	0.9 (2)	2.9 (3)	6.8 (18)	5.0 (16)	7.7 (37)	1.3 (6)	3.1 (5)	3.3 (12)	1.4 (6)	4.4 (12)
Don’t know/refused	0.0 (0)	0.0 (0)	0.0 (0)	0.0 (0)	0.0 (0)	17.7 (1)	3.0 (1)	13.1 (1)	35.4 (1)	0.0 (0)	0.0 (0)	0.0 (0)

© 2022 East KA et al. *JAMA Network Open.*

eTable 14. Number and Proportion of Past 30-Day Smokers Who Report Smoking Any Capsule (Not Menthol Only) Cigarettes in the Past 30 Days by Demographics, Frequent Smoking, Cigarettes Smoked per Day, Perceived Addiction, and Urges to Smoke, Split by Country and Survey Wave. Data are weighted % (unweighted n).

	CANADA				ENGLAND				US			
	Aug 2018 (N=584)	Aug 2019 (N=557)	Feb 2020 (N=614)	Aug 2020 (N=358)	Aug 2018 (N=634)	Aug 2019 (N=588)	Feb 2020 (N=936)	Aug 2020 (N=685)	Aug 2018 (N=445)	Aug 2019 (N=548)	Feb 2020 (N=630)	Aug 2020 (N=488)
**AGE GROUP**												
16-17	18.4 (44)	25.3 (44)	22.4 (59)	22.5 (39)	31.8 (55)	35.6 (76)	38.3 (135)	24.7 (55)	36.9 (77)	42.6 (90)	42.9 (127)	48.9 (119)
18-19	21.4 (73)	23.8 (76)	23.9 (76)	15.3 (27)	34.5 (155)	42.4 (171)	51.0 (292)	31.5 (154)	33.9 (80)	40.4 (138)	42.4 (122)	39.2 (91)
**SEX**												
Male	21.1 (67)	28.6 (59)	25.6 (61)	19.4 (31)	34.7 (88)	37.1 (81)	48.4 (176)	29.8 (87)	34.8 (75)	42.6 (94)	44.1 (109)	49.3 (114)
Female	18.1 (50)	18.7 (61)	20.5 (74)	18.0 (35)	31.4 (122)	42.3 (166)	42.6 (251)	27.1 (122)	36.2 (82)	39.5 (134)	40.3 (140)	33.2 (96)
**RACE/ETHNICITY**												
White only	14.0 (39)	22.4 (63)	18.5 (68)	16.4 (37)	32.7 (174)	38.7 (202)	46.1 (365)	27.8 (169)	34.3 (101)	39.2 (149)	42.5 (170)	43.5 (131)
Any black	35.8 (14)	27.9 (13)	47.6 (17)	28.4 (6)	37.5 (12)	43.8 (13)	49.2 (29)	38.0 (20)	34.6 (23)	58.5 (39)	45.4 (31)	43.1 (29)
Any other	27.1 (50)	28.4 (42)	29.2 (48)	22.9 (23)	36.2 (22)	45.3 (29)	39.2 (31)	27.8 (18)	40.3 (32)	44.3 (39)	41.8 (47)	50.8 (50)
Don't know/refused	16.5 (14)	16.2 (2)	22.6 (2)	0.0 (0)	41.5 (2)	30.1 (3)	42.1 (2)	61.3 (2)	53.9 (1)	26.3 (1)	30.9 (1)	0.0 (0)
**FREQUENT SMOKING**												
Other	20.5 (70)	25.1 (83)	23.1 (97)	20.1 (50)	30.8 (130)	34.2 (146)	41.3 (243)	27.0 (134)	33.3 (98)	41.2 (155)	38.9 (153)	39.5 (121)
≥20 of past 30 days	19.1 (47)	22.9 (37)	23.6 (38)	15.3 (16)	37.8 (80)	51.1 (101)	53.9 (184)	31.9 (75)	39.5 (59)	41.8 (73)	51.0 (96)	51.2 (89)
**CIGARETTES PER DAY**												
≤1	21.9 (53)	23.7 (56)	21.8 (60)	19.3 (34)	26.0 (75)	27.4 (68)	37.7 (145)	24.4 (75)	25.2 (49)	43.7 (97)	37.2 (95)	36.1 (72)
2 to 5	21.3 (41)	26.1 (37)	26.7 (56)	19.4 (21)	37.2 (90)	42.5 (97)	50.5 (154)	31.7 (73)	42.1 (61)	39.0 (74)	46.5 (93)	44.8 (74)
>5	15.6 (23)	24.3 (26)	22.2 (19)	16.9 (11)	41.2 (44)	57.7 (80)	54.2 (123)	34.3 (59)	48.2 (46)	40.9 (55)	48.5 (59)	54.9 (62)
Don't know/refused	0.0 (0)	16.3 (1)	0.0 (0)	0.0 (0)	17.1 (1)	31.3 (2)	34.9 (5)	10.7 (2)	32.5 (1)	21.7 (2)	28.7 (2)	23.1 (2)
**PERCEIVED ADDICTION**												
Not at all	15.7 (31)	22.7 (42)	14.0 (27)	13.5 (17)	23.8 (83)	27.3 (75)	37.3 (127)	18.2 (63)	21.4 (38)	33.7 (57)	27.7 (53)	32.0 (51)
A little/very	23.0 (86)	26.1 (78)	28.3 (106)	21.7 (47)	41.2 (127)	49.2 (167)	51.6 (298)	35.7 (143)	42.5 (116)	45.7 (170)	49.3 (191)	49.7 (156)
Don't know/refused	0.0 (0)	0.0 (0)	18.1 (2)	43.3 (2)	0.0 (0)	53.8 (5)	15.0 (2)	33.0 (3)	43.6 (3)	4.9 (1)	70.0 (5)	61.6 (3)
**URGES TO SMOKE**												
Less often	17.1 (50)	20.3 (54)	17.9 (54)	16.3 (32)	26.0 (103)	32.3 (118)	39.4 (183)	24.8 (98)	20.0 (40)	33.8 (72)	32.9 (85)	35.6 (74)
Every or most days	22.9 (66)	30.6 (65)	30.3 (81)	22.6 (34)	42.5 (106)	51.3 (128)	52.7 (241)	34.5 (109)	48.6 (114)	47.1 (156)	52.5 (162)	51.6 (136)
Don't know/refused	15.2 (1)	11.1 (1)	0.0 (0)	0.0 (0)	16.8 (1)	19.6 (1)	40.1 (3)	11.1 (2)	59.9 (3)	0.0 (0)	11.5 (2)	0.0 (0)

© 2022 East KA et al. *JAMA Network Open.*

eTable 15. Contrasts Within Countries for the Percentage of Past 30-Day Smokers Who Smoked Capsule (Not Menthol Only) Cigarettes by Survey Wave (N=7,067)

	Usual brand/variety of cigarettes is capsule (yes vs. other)			Smoked any capsule cigarettes in the past 30 days (yes vs. other)		
	**%**	**AOR (95% CI)**	**p**	**%**	**AOR (95% CI)**	**p**
**ENGLAND**						
Aug 2020	2.7	REF		28.6	REF	
Feb 2020	8.1	**1.02 (1.00-1.05)**	**.025**	45.6	1.05 (0.99-1.11)	.086
Aug 2019	5.1	**1.02 (1.00-1.04)**	**.035**	39.6	**1.12 (1.05-1.19)**	**<.001**
Aug 2018	5.3	**1.05 (1.03-1.08)**	**<.001**	33.3	**1.19 (1.13-1.25)**	**<.001**
**CANADA**						
Aug 2020	1.5	REF		18.8	REF	
Feb 2020	0.4	0.99 (0.97-1.01)	.369	23.2	1.01 (0.96-1.07)	.676
Aug 2019	1.2	1.00 (0.98-1.02)	.703	24.4	1.05 (0.99-1.12)	.084
Aug 2018	0.7	0.99 (0.97-1.01)	.177	19.9	1.05 (0.99-1.11)	.125
**US**						
Aug 2020	2.8	REF		44.1	REF	
Feb 2020	2.0	1.00 (0.98-1.03)	.752	42.6	**0.91 (0.85-0.99)**	**.023**
Aug 2019	2.9	1.00 (0.98-1.02)	.969	41.4	0.97 (0.90-1.05)	.507
Aug 2018	3.2	0.99 (0.97-1.01)	.471	35.3	0.98 (0.91-1.07)	.690

Data are weighted. Contrasts (AOR, 95% CI, p) are derived from interactions from logistic regression models adjusted for age group, sex, and race/ethnicity.

© 2022 East KA et al. *JAMA Network Open.*

eTable 16. Contrasts Within Countries for the Percentage of Past 30-Day Smokers Who Smoked Capsule (Not Menthol Only) Cigarettes by Demographics (N=7,067)

	Usual brand/variety of cigarettes is capsule (yes vs. other)			Smoked any capsule cigarettes in the past 30 days (yes vs. other)		
	**%**	**AOR (95% CI)**	**p**	**%**	**AOR (95% CI)**	**p**
**CANADA**						
AGE						
16-17	1.0	REF		22.0	REF	
18-19	0.9	1.00 (0.99-1.01)	.849	21.4	0.99 (0.95-1.03)	.566
SEX						
Male	1.3	REF		23.7	REF	
Female	0.4	0.99 (0.98-1.00)	.071	18.9	**0.95 (0.91-0.99)**	**.007**
RACE/ETHNICITY						
White only	1.3	REF		18.1	REF	
Any black	0.0	- [1]		34.7	**1.18 (1.08-1.28)**	**<.001**
Any other or mixed	0.7	0.99 (0.98-1.00)	.224	26.9	**1.10 (1.05-1.15)**	**<.001**
Don't know/refused[1]	0.0	- [1]	-	15.8	0.99 (0.92-1.07)	.808
**ENGLAND**						
AGE						
16-17	4.5	REF		32.8	REF	
18-19	6.2	1.02 (1.00-1.03)	.072	40.6	**1.08 (1.04-1.12)**	**<.001**
SEX						
Male	4.4	REF		38.1	REF	
Female	6.9	**1.02 (1.00-1.04)**	**.014**	36.3	0.98 (0.94-1.02)	.261
RACE/ETHNICITY						
White only	5.9	REF		36.9	REF	
Any black	2.5	**0.97 (0.95-0.99)**	**.001**	42.4	1.05 (0.97-1.15)	.240
Any other or mixed	3.6	0.98 (0.95-1.00)	.088	37.4	1.00 (0.93-1.07)	.980
Don't know/refused	8.1	1.02 (0.91-1.14) [2]	.706	40.5	1.03 (0.83-1.28) [2]	.786
**US**						
AGE						
16-17	2.7	REF		42.5	REF	
18-19	2.8	1.00 (0.98-1.02)	.994	38.6	0.96 (0.91-1.02)	.163
SEX						
Male	1.6	REF		42.4	REF	
Female	4.7	**1.03 (1.01-1.05)**	**.003**	37.3	**0.95 (0.90-1.00)**	**.041**
RACE/ETHNICITY						
White only	3.1	REF		39.7	REF	
Any black	2.0	0.99 (0.97-1.01)	.322	43.8	1.04 (0.96-1.13)	.306
Any other or mixed	1.4	**0.98 (0.97-1.00)**	**.028**	43.4	1.03 (0.97-1.11)	.337
Don't know/refused	0.0	- [1,2]		40.0	1.00 (0.63-1.58) [2]	.995

Data are weighted. Contrasts (AOR, 95% CI, p) are derived from interactions from logistic regression models adjusting for country, survey wave, age, sex, and race/ethnicity. Data are aggregated across survey waves. [1] Estimate unreportable due to n=0. [2] Treat estimate with caution (denominator n<30).

© 2022 East KA et al. *JAMA Network Open.*

eTable 17. Contrasts Within Countries for the Percentage of Past 30-Day Smokers Who Smoked Capsule (Not Menthol Only) Cigarettes by Consumption/Dependence Indicators (N=7,067)

	Usual brand/variety of cigarettes is capsule (yes vs. other)			Smoked any capsule cigarettes in the past 30 days (yes vs. other)		
	%	AOR (95% CI)	p	%	AOR (95% CI)	p
**CANADA**						
FREQUENT SMOKING						
Other	0.9	REF		22.2	REF	
≥20 of past 30 days	1.1	1.00 (0.99-1.02)	.747	20.4	0.99 (0.95-1.03)	.500
CIGARETTES PER DAY						
≤1	0.8	REF		21.7	REF	
2-5	1.0	1.00 (0.99-1.01)	.799	23.4	1.02 (0.97-1.06)	.421
>5	1.2	1.00 (0.99-1.02)	.612	19.7	0.99 (0.94-1.04)	.596
Don't know/refused[1]	0.0	- [1,2]	-	3.9	**0.84 (0.78-0.91)** [2]	**<.001**
PERCEIVED ADDICTION						
Not at all	0.1	REF		16.6	REF	
A little/very	1.4	**1.01 (1.01-1.02)**	**.002**	24.8	**1.08 (1.04-1.13)**	**<.001**
Don't know/refused[1]	0.0	-[1,2]		13.1	0.96 (0.85-1.08) [2]	.470
URGES TO SMOKE						
Less often	0.5	REF		17.9	REF	
Every or most days	1.5	1.01 (1.00-1.02)	.059	26.5	**1.09 (1.05-1.14)**	**<.001**
Don't know/refused[1]	0.0	- [1,2]		7.0	0.90 (0.82-0.99) [2]	.030
**ENGLAND**						
FREQUENT SMOKING						
Other	5.6	REF		33.8	REF	
≥20 of past 30 days	5.3	1.00 (0.98-1.01)	.687	44.1	**1.11 (1.07-1.16)**	**<.001**
CIGARETTES PER DAY						
≤1	4.3	REF		29.6	REF	
2-5	7.2	**1.03 (1.01-1.05)**	**.013**	41.1	**1.12 (1.07-1.18)**	**<.001**
>5	5.6	1.01 (0.99-1.03)	.313	47.1	**1.19 (1.13-1.26)**	**<.001**
Don't know/refused	0.0	- [1]		24.7	0.95 (0.82-1.09)	.437
PERCEIVED ADDICTION						
Not at all	5.3	1.00		27.1	REF	
A little/very	5.6	1.01 (0.99-1.02)	.544	44.8	**1.19 (1.15-1.24)**	**<.001**
Don't know/refused	3.9	0.99 (0.92-1.07) [2]	.790	31.1	1.04 (0.86-1.25) [2]	.687
URGES TO SMOKE						
Less often	5.4	1.00		31.0	REF	
Every or most days	5.5	1.00 (0.98-1.02)	.782	45.6	**1.16 (1.11-1.21)**	**<.001**
Don't know/refused	8.8	1.04 (0.92-1.16) [2]	.548	21.4	0.92 (0.77-1.09) [2]	.331
**US**						
FREQUENT SMOKING						
Other	3.0	REF		37.8	REF	
≥20 of past 30 days	2.2	0.99 (0.97-1.01)	.416	45.8	**1.09 (1.03-1.16)**	**.003**
CIGARETTES PER DAY						
≤1	1.6	REF		34.4	REF	
2-5	3.2	1.02 (1.00-1.03)	.058	43.3	**1.09 (1.03-1.16)**	**.005**
>5	3.8	1.02 (0.99-1.06)	.138	48.8	**1.16 (1.08-1.25)**	**<.001**
Don't know/refused	15.1	1.15 (0.89-1.49) [2]	.274	27.9	0.94 (0.71-1.25) [2]	.685
PERCEIVED ADDICTION						
Not at all	2.9	REF		27.9	REF	
A little/very	2.5	1.00 (0.98-1.02)	.802	46.6	**1.21 (1.14-1.27)**	**<.001**
Don't know/refused	14.5	1.13 (0.92-1.39) [2]	.233	44.5	1.21 (0.95-1.53) [2]	.126
URGES TO SMOKE						
Less often	2.1	REF		29.6	REF	
Every or most days	3.1	1.01 (0.99-1.03)	.205	49.9	**1.23 (1.16-1.30)**	**<.001**
Don't know/refused	16.0	1.14 (0.89-1.46) [2]	.311	30.8	1.02 (0.75-1.40) [2]	.880

Data are weighted. Contrasts (AOR, 95% CI, p) are derived from interactions from logistic regression models adjusting for country, survey wave, age, sex, and race/ethnicity. Data are aggregated across survey waves.
[1] Estimate unreportable due to n=0. [2] Treat estimate with caution (denominator n<30).

© 2022 East KA et al. *JAMA Network Open.*

eTable 18. Adjusted Logistic Regression Models Predicting Capsule (Not Menthol Only) Cigarette Smoking From Survey Wave, Country, and Demographic Covariates

		Usual brand/variety of cigarettes is capsule (yes vs. other)			Smoked any capsule cigarettes in the past 30 days (yes vs. other)		
	**Sample n**	**%**	**AOR (95% CI)**	**p**	**%**	**AOR (95% CI)**	**p**
**SURVEY WAVE**							
Aug 2020	1531	2.4	REF		30.2	REF	
Feb 2020	2180	4.9	1.93 (1.25-2.98)	**.003**	39.6	**1.53 (1.29-1.81)**	**<.001**
Aug 2019	1693	3.3	1.44 (0.90-2.29)	.125	35.3	**1.32 (1.10-1.58)**	**.002**
Aug 2018	1663	3.5	1.5 (0.92-2.45)	.104	30.5	1.00 (0.84-1.20)	.968
**COUNTRY**							
Canada	2113	0.9	REF		21.6	REF	
England	2843	5.5	**5.33 (2.93-9.70)**	**<.001**	37.3	**2.25 (1.94-2.62)**	**<.001**
US	2111	2.8	**2.89 (1.49-5.61)**	**.002**	40.5	**2.58 (2.19-3.05)**	**<.001**
**AGE GROUP**							
16-17	2786	3.1	REF		32.8	REF	
18-19	4281	3.9	1.26 (0.92-1.74)	.155	35.0	1.10 (0.98-1.25)	.117
**SEX**							
Male	2,938	2.8	REF		35.6	REF	
Female	4,129	4.6	**1.61 (1.17-2.22)**	**.003**	31.8	**0.84 (0.74-0.94)**	**.003**
**RACE/ETHNICITY**							
White only	5,019	4.2	REF		33.8	REF	
Any black	607	1.6	**0.43 (0.22-0.82)**	**.011**	40.7	**1.42 (1.15-1.76)**	**.001**
Any other or mixed	1,284	1.7	**0.55 (0.33-0.93)**	**.025**	33.5	**1.24 (1.05-1.46)**	**.010**
Don't know/refused	157	1.6	0.76 (0.16-3.52)	.727	21.8	0.91 (0.58-1.43)	.678

All data except sample n are weighted.

© 2022 East KA et al. *JAMA Network Open.*

eFigure 7. Percentage of Past 30-Day Smokers Who Reported a Usual Brand/Variety of Cigarettes That Was Capsule (Not Menthol Only) Cigarettes by Demographic Characteristics in England at Each Survey Wave (N=2,843)

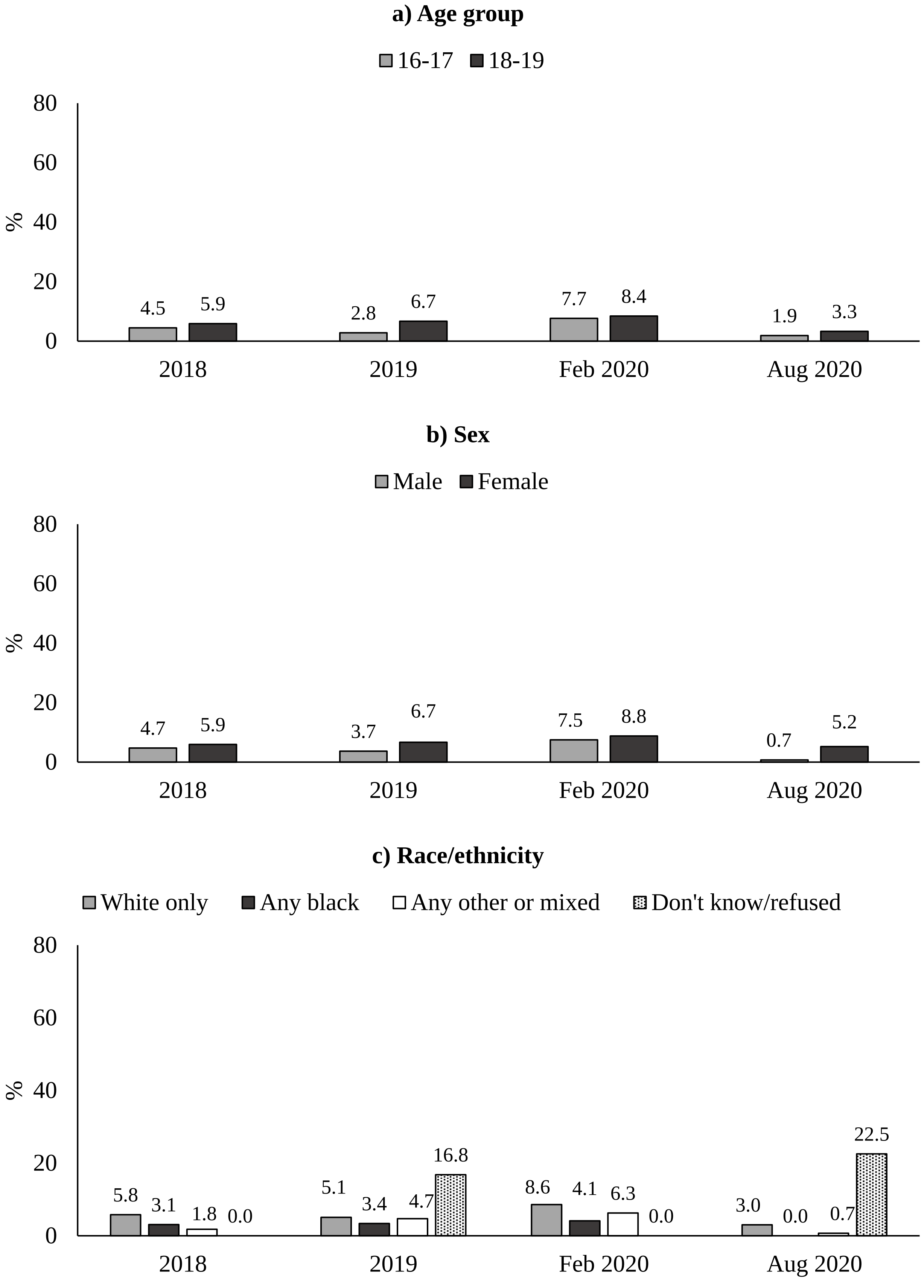

© 2022 East KA et al. *JAMA Network Open.*

eFigure 8. Percentage of Past 30-Day Smokers Who Reported That They Had Smoked Any Capsule (Not Menthol Only) Cigarettes in the Past 30 Days by Demographic Characteristics in England at Each Survey Wave (N=2,843)

a) Age group

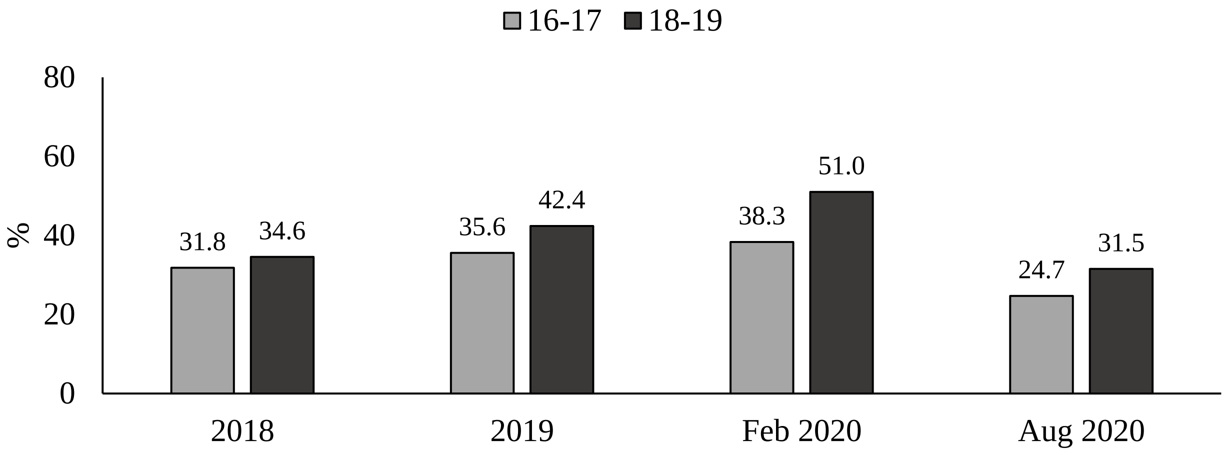

b) Sex

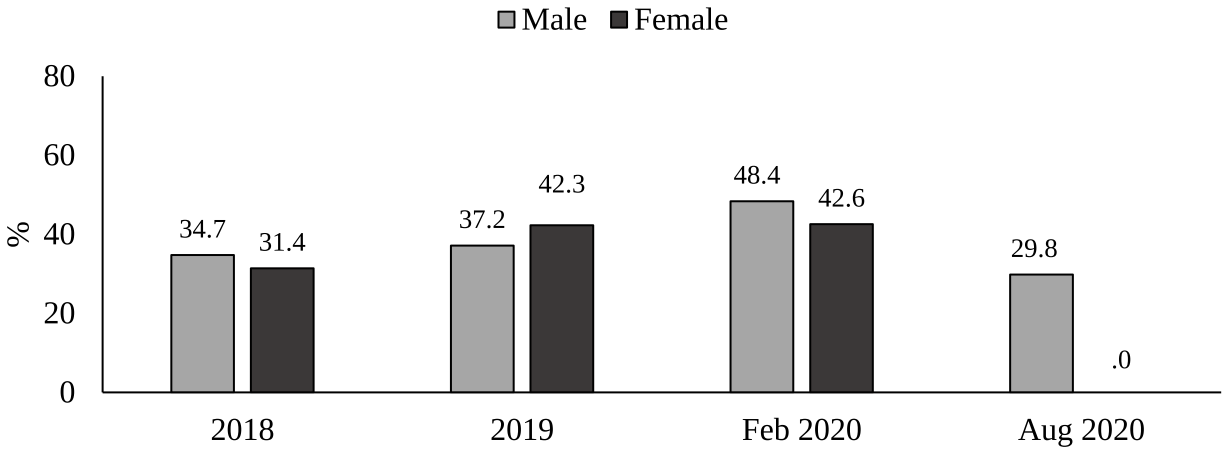

c) Race/ethnicity

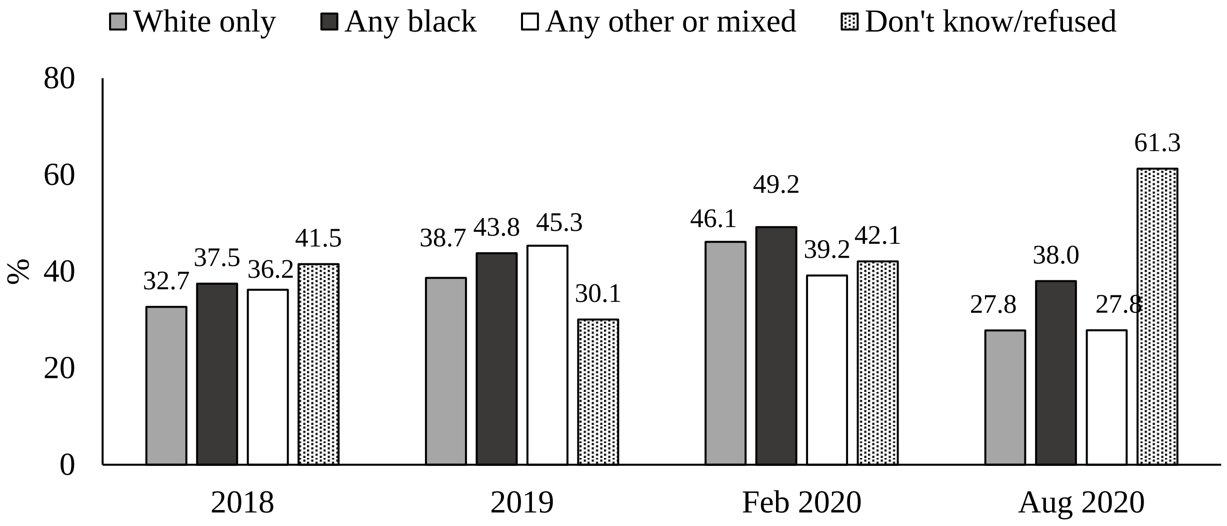

© 2022 East KA et al. *JAMA Network Open.*

eFigure 9. Percentage of Past 30-Day Smokers Who Reported a Usual Brand/Variety of Cigarettes That Was Capsule (Not Menthol Only) by Consumption/Dependence Indicators in England at Each Survey Wave (N=2,843)

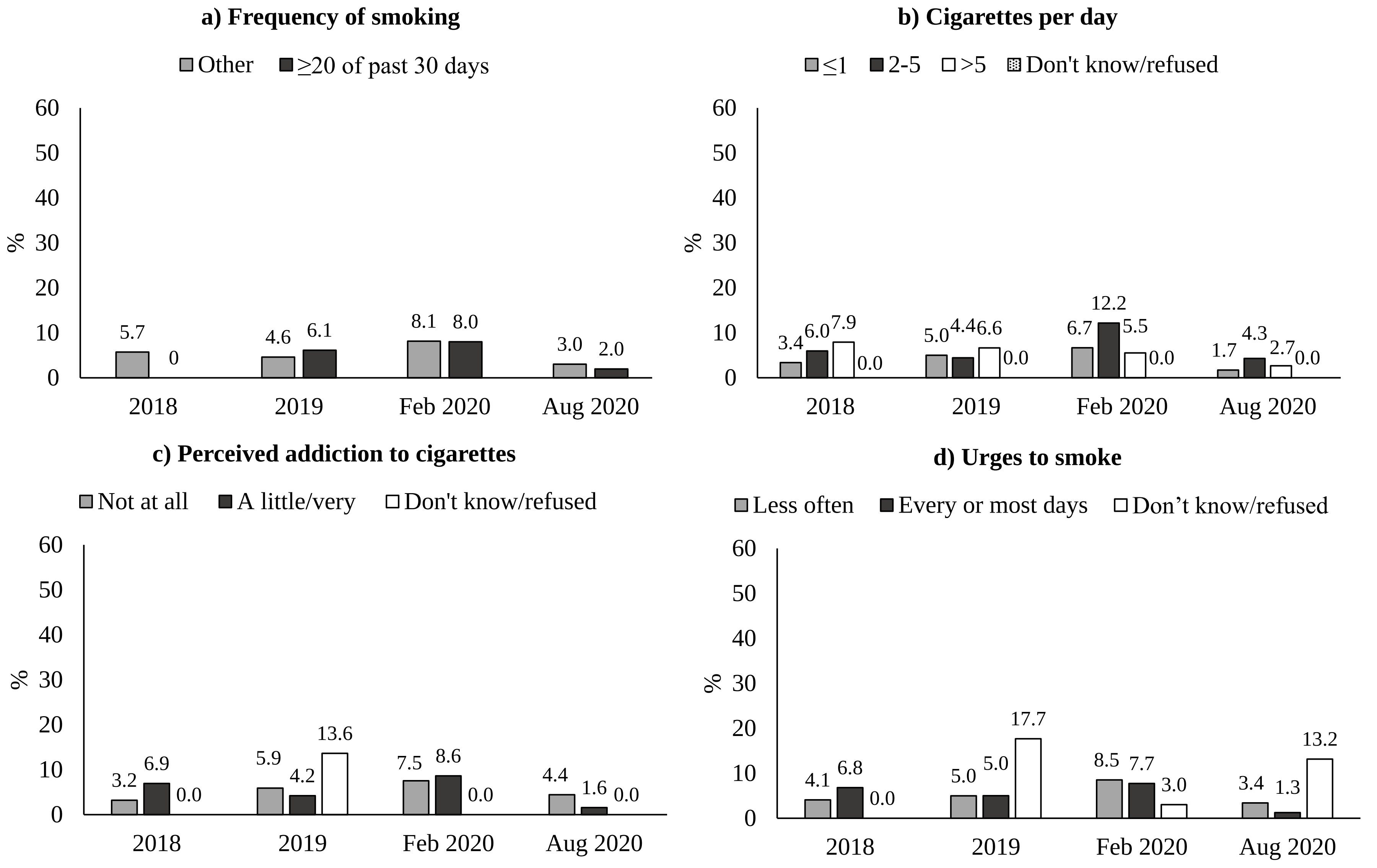

© 2022 East KA et al. *JAMA Network Open.*

eFigure 10. Percentage of Past 30-Day Smokers Who Reported That They Had Smoked Any Capsule (Not Menthol Only) Cigarettes in the Past 30 Days by Consumption/Dependence Indicators in England at Each Survey Wave (N=2,843)

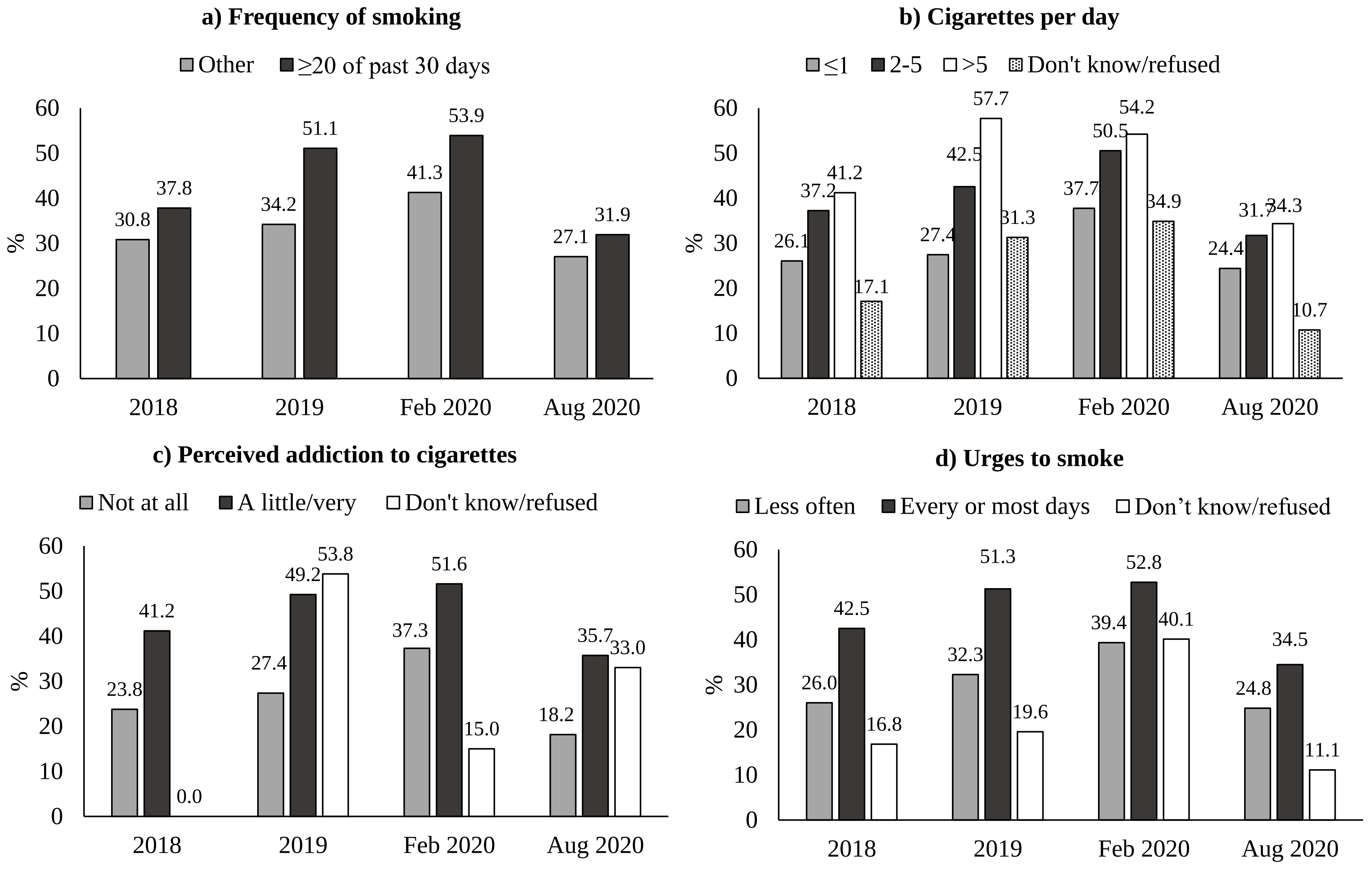

© 2022 East KA et al. *JAMA Network Open.*
